# *Babesia behnkei* sp. nov., a novel *Babesia* species infecting isolated populations of Wagner’s gerbil, *Dipodillus dasyurus,* from the Sinai Mountains, Egypt

**DOI:** 10.1186/s13071-014-0572-9

**Published:** 2014-12-09

**Authors:** Anna Bajer, Mohammed Alsarraf, Małgorzata Bednarska, Eman ME Mohallal, Ewa J Mierzejewska, Jolanta Behnke-Borowczyk, Sammy Zalat, Francis Gilbert, Renata Welc-Falęciak

**Affiliations:** Department of Parasitology, Institute of Zoology, Faculty of Biology, University of Warsaw, 1 Miecznikowa Street, 02-096 Warsaw, Poland; Desert Research Center, Cairo, Egypt; Department of Forest Phytopathology, Faculty of Forestry, Poznań University of Life Sciences, Poznań, Poland; Department of Zoology, Suez Canal University, Ismailia, Egypt; Faculty of Medicine & Health Sciences, School of Biology, University of Nottingham, Nottingham, UK

**Keywords:** *Babesia*, *Dipodillus dasyurus*, 18S rDNA, ITS2, Phylogenetic analysis, Sinai, Egypt

## Abstract

**Background:**

Although a number of new species of *Babesia/Theileria* have been described recently, there are still relatively few reports of species from Africa. In this study based on the evaluation of morphology and phylogenetic relationships, we describe a novel species from Wagner’s gerbil, *Babesia behnkei* n. sp.

**Methods:**

Rodents (n = 1021) were sampled in four montane valleys (wadies) in 2000, 2004, 2008 and 2012 in the Sinai Mountains, Egypt. The overall prevalence of *Babesia* spp. was highest in the Wagner’s gerbil (*Dipodillus dasyurus*; 38.7%) in comparison to the prevalence in the spiny mice species, *Acomys dimidiatus* and *A. russatus*. Morphological investigations were conducted for the comparison of trophozoites of the novel species of *Babesia* with the *B. microti* King’s 67 reference strain. Thirty-two isolates derived from *D. dasyurus* over a 9 year period (2004-2012) from two wadies (29 isolates from Wadi Gebel and 3 from Wadi El-Arbaein) were investigated by microscopic, molecular and phylogenetic analysis. A near-full-length sequence of the 18S rRNA gene and the second internal transcribed spacer (ITS2) region were amplified, sequenced and used for the construction of phylogenetic trees.

**Results:**

A novel species of *Babesia* was identified in two isolated populations of *D. dasyurus*. Phylogenetic analysis of 18S rDNA and ITS2 sequences revealed that *B. behnkei* n. sp. is most closely related to *B. lengau* from cheetahs from South Africa and to Nearctic species found only in North America (the pathogenic *B. duncani* and *B. conradae*) and that it is more distant to the cosmopolitan rodent parasite *B. microti.* Trophozoites of *B. behnkei* were smaller and less polymorphic than trophozoites of *B. microti*.

**Conclusion:**

*Babesia behnkei* n. sp. is a novel species of the ‘Duncani group’ maintained in isolated populations of *Dipodillus dasyurus* occurring in the Sinai Mountains of Egypt.

**Electronic supplementary material:**

The online version of this article (doi:10.1186/s13071-014-0572-9) contains supplementary material, which is available to authorized users.

## Background

The genus *Babesia* comprises tick-transmitted, intraerythrocytic protozoan parasites of many different vertebrates including humans [1,2]. Currently there are over 120 recognized species of *Babesia* described from various parts of the world. Even in the last two decades new species have been added to the list, e.g. *B. venatorum* in humans in Europe, *B. benneti* in the yellow-legged gull [3], *B. hongkongensis* in feral cats in Hongkong [4] and a novel *Babesia/Theileria* species from marsupials in Australia [5].

In contrast to the rest of the world, relatively few new species have been described from African hosts in recent years, including for example *B. lengau* from cheetahs [6], *B. bicornis* from black rhinoceros [7], *B. ugwidiensis* from cormorants [8] and *B. leo* from lions [9]. Additionally, putative new species of *Babesia/Theileria* have been reported from sable antelopes [10] and wild felids from Kenya [11]. It is pertinent that new species of *Babesia* (and presumably also other haemoparasites) are often discovered at post-mortem examinations, especially in the case of endangered host species such as the sable antelope and the black rhinoceros.

The diversity of *Babesia* spp. depends on many factors, including host-parasite or vector-parasite specificities, well reflected in the geographically restricted distribution of some species. Cosmopolitan species include parasites of livestock and horses (*B. bovis, B. divergens, B. equi, B. ovis*), dogs or cats (*B. canis, B. rossi, B. vogeli, B. felis*) or rodents (*B. microti*). Other species are specific to particular hosts whose distribution is restricted to continents, as for example with *B. conradae, B. duncani* or *B. odocoilei* found in North America, *B. benetti, B. capreoli, B. venatorum* found in Europe, and *B. crassa, B. hongkongensis, B. motasi, B. orientalis* found only in Asia. In this last Asian group of species, some have been identified to date only in a single host species. However, it is also well established that some hosts are susceptible to, and can carry concurrently, more than a single species of *Babesia/Theileria*; often these species are indistinguishable by conventional microscopy. For example, cats are susceptible to infection with *B. felis* but also with *B. leo*, *B. hongkongensis* and *B. lengau* [4,12,13]. In view of this complexity, it is highly likely that many *Babesia* spp. remain still unrecognized, especially those infecting rarely studied wild species of hosts in isolated regions of the world.

Conventionally and historically, new species of *Babesia* have been erected based on their hosts and on morphological criteria. However, the trophozoites of different species of ‘small’ *Babesia* and *Theileria* spp. in erythrocytes appear very similar under light microscopy and their differentiation is difficult. In recent decades however, the use of molecular tools have made a significant impact on the field and the sequencing of selected gene fragments has greatly improved the accuracy and reliability of species identification. However, because of the morphological similarities, the systematics of *Babesia/Theileria* spp. are still not fully resolved and in urgent need of revision in view of the many recently conducted molecular phylogenetic studies [14-16]. Based on these, the distant clades of ‘*Babesia*’, including some species that were misidentified as ‘*Theileria*’ [14], require revision of their generic status and new nomenclature. Thus the use of molecular tools, which are clearly more sensitive than conventional morphology based on light microscopy, remains crucial for distinguishing between and for the identification of *Babesia/Theileria* spp. and for their assignment to particular clades.

One cosmopolitan species of public health concern is *B. microti*, the main cause of human babesiosis in the United States of America [2,17,18] but also identified recently in humans in Europe [19,20], China and Japan [21]. This species had been originally described as *Smithia microti* in Portugal from the vole *Microtus incertus* [22]; voles of the genus *Microtus* are still considered to be the main reservoir of this parasite worldwide [22,23]. Surprisingly *B. microti* has subsequently been found in a wide variety of rodent species worldwide [21,22,24-34].

In Eurasia and North America the main rodent hosts of *B. microti* are different species of voles, *Microtus* spp.*, Myodes (Clethrionomys*) spp. and mice, *Apodemus* spp*.* At least 8 species of *Microtus* have been reported as commonly infected with *B. microti*: *M. arvalis*, *M. agrestis* and *M. oeconomus* from Europe [22,32,35,36], *M. montebelli* from Japan and *M. miurus*, *M. montanus*, *M. ochrogaster* and *M. pennsylvanicus* from North America [21,33,34,37]. Additionally, 4 species of *Myodes* (*Clethrionomys*) (*M. glareolus*, *M. gapperi*, *M. rufocanus* and *M. rutilus*) and 5 species of *Apodemus (A. agrarius*, *A. argenteus*, *A. flavicollis*, *A. speciosus* and *A. sylvaticus)* have been reported as hosts of *B. microti* worldwide. *Peromyscus leucopus* has recently been shown to act as a competent host in North America [27] and infected *P. keeni* have been reported from Alaska [37]. Other species of rodents reported to host *B. microti* include eastern chipmunks *Tamias striatus* [27]. However, carnivores (i.e. foxes, raccoons) and insectivores such as shrews (at least 5 species of *Sorex*, *Blarina* and others) may also serve as hosts of *B. microti* [21,25,27,33-35]. On the basis of the above, *B. microti* appears to be the most widely distributed species worldwide evidently lacking tight host-specificity, but caution is warranted. Among the many studies on rodent haemoparasites reporting the presence of infections with *B. microti* [38-42], it is suspected that few have appropriately and critically assessed the species identity; rather it has been merely assumed that the parasite is *B. microti* because it was detected in a rodent host. In fact, recent studies have shown that at least three distinct clades, differing in their host-specificity, exist among isolates of *B. microti* that have been genotyped [43,44]. Another species of *Babesia* infecting rodents, *B. rodhaini*, has been used worldwide as a laboratory model in mice and rats; this species seems to be closely related to the ‘Microti group’ according to phylogentic analysis [14].

Our research on the parasite fauna of wild rodents from the Sinai Mountains began in 2000, when we were invited by Professor Jerzy M. Behnke from the University of Nottingham to join an expedition of the university assessing the helminth communities of wild rodents in four isolated montane valleys. Initially, the study focused on gastrointestinal parasites of *Acomys dimidiatus*, the most abundant rodent species inhabiting Bedouins’ gardens [45,46]. Subsequently, intestinal protozoa and haemoparasites were incorporated into the study [38] and the host range extended. The highest prevalence of infections with *Babesia* and *Bartonella* spp. were found in the Wagner’s gerbil, *Dipodillus dasyurus*, one of the three most numerous rodent species in the study sites (unpublished data). Primary molecular research on the *Babesia*-positive samples revealed surprisingly low homology (approx. 96%) of partial (550 bp) 18S rDNA sequence to those for *B. microti* and other named species. Therefore, exploiting material collected in the latter expeditions to the study sites, we characterized this novel species of *Babesia* by light microscopy study and molecular and phylogenetic analyses.

## Methods

### Field studies in Sinai, Egypt

Fieldwork was conducted over 4-5-week periods in August-September 2000, 2004, 2008 and 2012 and was based at the Environmental Research Centre of Suez Canal University (2000, 2004) or at Fox Camp (2008, 2012) in the town of St Katherine, South Sinai, Egypt. Trapping was carried out in four montane wadis (dry valleys) in the vicinity of St Katherine. The local environment and general features of the four study sites (Wadi El-Arbaein, Wadi Gebel, Wadi Itlah and Wadi Gharaba), as well as their spatial relationships with one another, have been described elsewhere [46]. At each site, rodents were caught live in Sherman traps, placed selectively among the rocks and boulders around walled gardens and occasionally along the lower slopes of wadis. These were set out at dusk, and inspected in the early morning before exposure to direct sunlight. All traps were brought to the local or main camp, where the animals were identified and processed. Traps were re-set the following evening.

**Figure 1:**
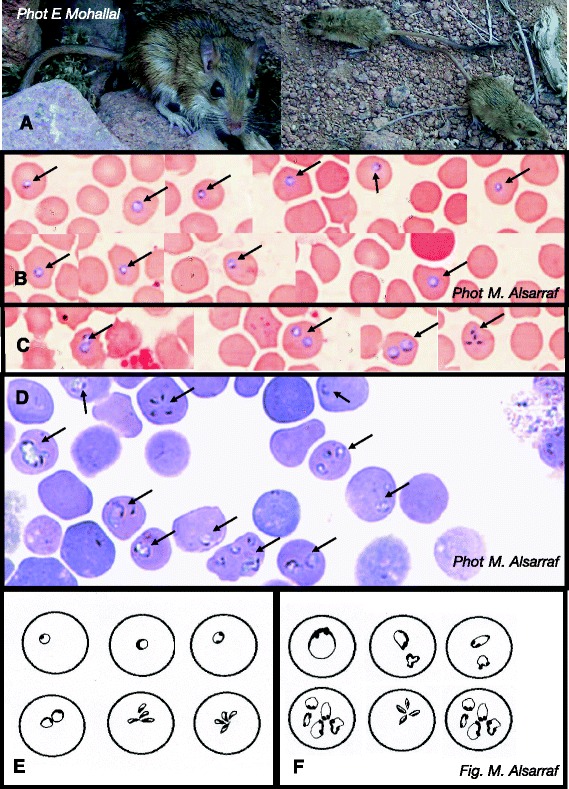
**The type-host, Wagner’s gerbil** ***Dipodillus dasyurus*** **(W. Gebel) trapped in Sinai, Egypt, and type-forms of** ***Babesia behnkei*** **n. sp. A**. Type host: Wagner’s gerbil, *Dipodillus dasyurus* (W. Gebel, Sinai, Egypt). **B**. Type-forms of *Babesia behnkei* n. sp. ex Wagner’s gerbil *Dipodillus dasyurus* (W. Gebel) collected in Sinai, Egypt. Typical forms - single rounded trophozoites in erythrocytes. **C**. Double trophozoites and dividing form (tetrad) of *Babesia behnkei* n. sp. ex Wagner’s gerbil *Dipodillus dasyurus* in erythrocytes. **D**. Trophozoites of *Babesia microti* King’s 67 in erythrocytes of BALB/c mice (acute phase, on the 8^th^ day post infection). **E**. Different forms of *Babesia behnkei* n. sp. ex *D. dasyurus*. **F**. Different forms of *Babesia microti* from BALB/c mice.

The three most abundant rodent species (*A. dimidiatus*, *A. russatus* and *D. dasyurus*) (Table 1) were sampled- sexed, weighed, measured and scrutinized for obvious lesions as described by [46]. Ectoparasites observed during field examination were removed and placed in 70% ethanol. Blood and faecal samples were taken and animals were then either fur marked individually and released close to the point of capture (Figure 1A), or returned to the main camp at St Katherine for autopsy. A maximum of 40% of the captured rodents from each site were culled (by agreement with the St Katherine National Protectorate authorities).

**Table 1:** **Structure of the rodent communities sampled and numbers of hosts studied during 2000-2012**

**Year of study**	**Host species**	**Site (wadi)**				**No. of rodents**	
		**W. El Arbaein**	**W. Gebel**	**W. Gharaba**	**W. Itlah**	**Total by species**	**Total by year**
2000	*Acomys dimidiatus*	58	28	28	46	160	
	*Acomys russatus*	4	4	1	6	15	
	*Dipodillus dasyurus*	3	6	2	2	13	188
2004	*Acomys dimidiatus*	43	43	60	70	216	
	*Acomys russatus*	1	8	3	8	20	
	*Dipodillus dasyurus*	4	16	7	0	27	263
2008	*Acomys dimidiatus*	66	43	52	80	241	
	*Acomys russatus*	3	6	3	8	20	
	*Dipodillus dasyurus*	2	15	2	0	19	280
2012	*Acomys dimidiatus*	64	46	52	58	220	
	*Acomys russatus*	0	7	2	9	18	
	*Dipodillus dasyurus*	14	22	16	0	52	290
Total by site	*Acomys dimidiatus*	231	160	192	254	837	
	*Acomys russatus*	8	25	9	31	73	
	*Dipodillus dasyurus*	23	59	27	2	111	
Overall	Total no. of rodents	262	244	228	287		1021

### Blood collection and DNA extraction

Thin blood smears were prepared from drops of blood taken from the heart or tail tip. Blood smears were air-dried, fixed in absolute methanol and stained for 1 h in Giemsa stain in buffer at pH 7.2. In 2004, 2008 and 2012, in addition to blood smears, molecular techniques were used for the detection of *Babesia* spp*.* Blood from the tail vein was collected on FTA classic cards (Whatman, UK) for the long-time preservation of DNA. From the culled animals, 200 μl of whole blood were also collected into 0.001 M EDTA and frozen at -20°C. Genomic DNA was extracted from whole blood using DNAeasy Blood & Tissue kit (Qiagen, USA) or AxyPrep MiniPrep Blood kit (AxyGen, USA) and stored at -20°C. DNA from FTA cards was cleaned with FTA purification Reagent (Whatman, UK) accordingly to manufacturer’s instructions.

### Molecular characterization

Detection and genotyping of 32 *Babesia* isolates (Table 2) were performed by amplification and sequencing of ITS1, ITS2 and 18S rRNA regions/genes. The primers and thermal profiles used in this study have been described previously [7,10,47-50]. Reactions were performed in 1× PCR buffer, 1 U Taq polymerase, 1 μM of each primer and 2–5 μl of the extracted DNA sample. Negative controls were performed in the absence of template DNA. Primers GF (5’-G(C/T) (C/T)T TGT AAT TGG AAT GAT GG-3’) and GR (5’-CCA AAG ACT TTG ATT TCT CTC-3’) were used for the detection of *Babesia/ Theileria* spp. by the amplification of a 559 bp fragment of the 18S rDNA [48,49]. Primers Nbab_1F (5’-AGC CAT GCA TGT CTA AGT ATA AGC TTT T-3’) [10] and TB Rev (5’-AAT AAT TCA CCG GAT CAC TCG-3’) [50] were used for the genetic characterization of positive isolates by the amplification of a 1,700 bp near-full-length sequence of the 18S rRNA gene. As a second genetic marker, the 315 bp of the ITS2 region were amplified using the primers ITS2-F (5’-GGC TCA CAC AAC GAT GAA GG-3’) and ITS2-R (5’-CTC GCC GTT ACT AAG GGA ATC-3’) [7,47]. Additionally, a 615 bp sequence of the 18S-ITS1-5.8S region was amplified using the primers ITS1-F (5’-CGA GTG ATC CGG TGA ATT ATT C-3’) and ITS1-R (5’-CCT TCA TCG TTG TGT GAG CC-3’) [7,47]. PCR products were subjected to electrophoresis on a 1.5% agarose gel, stained with Midori Green stain (Nippon Genetics, GmbH) and sequenced by a private company (Genomed S.A., Poland).

**Table 2:** **Origin of the isolates of** ***B. behnkei*** **n. sp. from** ***Dipodillus dasyurus*** **used for genotyping and phylogenetic analysis**

**Year of study**	**W. El-Arbaein**	**W. Gebel**	**W. Gharaba**	**W. Itlah**	**All sites**
2004	0 isolates	12 isolates	nd	nd	12 isolates (W. Gebel)
2008	1 isolate	3 isolates	nd	nd	4 isolates (1 isolate W. El Arbaein, 3 isolates W. Gebel)
2012	2 isolates	14 isolates	nd	nd	16 isolates (2 isolates W. El Arbaein, 14 isolates W. Gebel)
Total	3 isolates	29 isolates	nd	nd	32 (3 isolates W. El Arbaein, 29 isolates W. Gebel)

Nd- not done, no isolates available.

### Sequence analysis

DNA sequence alignments and phylogenetic analyses were conducted using MEGA v. 6.0 [51]. Akaike information criterion was used in jModel Test to identify the most appropriate model of nucleotide substitution. Tamura 3-parameter (I + G) model was chosen as the most appropriate for the Maximum Likelihood analysis of the 18S rDNA alignment. Neighbor-Joining method was used as the tree construction method for ITS2 (MEGA v. 6.0), with Kimura 2-parameter model.

Sequences of species/strains of *Babesia*, *Theileria* and *Cytauxzoon* obtained from GenBank (www.ncbi.nlm.nih.gov) were used in the sequence alignment. The stability of inferred phylogenies was assessed by bootstrap analysis of 1,000 randomly generated sample trees.

### Morphology by light microscopy

Giemsa stained blood smears were examined under oil immersion (at × 1000 magnification). Parasites (*Babesia* spp., *Bartonella* spp., *Haemobartonella* (*Mycoplasma)* spp., *Hepatozoon* spp. and *Trypanosoma* spp.) were counted in 200 fields of vision. For comparison, stained blood smears prepared from BALB/c mice infected with *B. microti* King’s 67 strain were also examined [52]. Trophozoites of *Babesia* spp. were measured with a Nikon screw micrometer calibrated against a standard stage micrometer. Images of the novel *Babesia* forms were made with a digital camera integrated with Nikon Eclipse E600. Typical forms, characteristic of the isolates were drawn on the basis of more than 100 images.

### Statistical analysis

Quantitative data reflecting the mean diameter of trophozoites were compared between *B. behnkei* n. sp. and *B. microti* King’s 67 strain. The mean diameters were analyzed by multifactorial ANOVA with SPSS v. 21 using models with normal errors.

### Ethical issue

Rodents from St Katherine National Protectorate were sampled by agreement with the St Katherine National Protectorate authorities obtained for each set of field work. *B. microti* strain King’s 67, originally obtained from Dr. S. Randolph (Oxford University) is maintained in our laboratory by weekly blood passage in adult BALB/c females. Blood sampling was carried out in strict accordance with the recommendations in the Guide for the Care and Use of Laboratory Animals of National Ethics Committee for Animal Experimentation, Poland. The protocol no 214/2011 was approved by First Warsaw Local Ethics Committee for Animal Experimentation.

## Results

### Taxonomic review

*Babesia behnkei* n. sp.

*Type-host*: Wagner’s gerbil, *Dipodillus dasyurus* (Rodentia, Muridae, Gerbillinae).

*Type-locality*: Wadi Gebel in Sinai Mountains, Egypt.

*Other localities*: Wadi El-Arbaein in Sinai Mountains, Egypt.

*Type-material*: Hapanotype. Eg085 from *Dipodillus dasyurus,* sampled on 22 August 2004 in Wadi Gebel, Sinai Mountains, Egypt, deposited at the Natural History Museum, London, UK (NHMUK 2014.8.26.1).

Parahapanotypes. Eg083 (NHMUK 2014.8.26.2), Eg084 (NHMUK 2014.8.26.3) from *D. dasyurus*, sampled on 22 August 2004 in Wadi Gebel, Sinai Mountains, Egypt; Eg041(NHMUK 2014.8.26.4) from *D. dasyurus*, sampled on 17 August 2008, W. El-Arbaein, Sinai Mountains, Egypt; Eg026 (NHMUK 2014.8.26.5), Eg028 (NHMUK 2014.8.26.6) from *D. dasyurus* sampled on 17 August 2012, W. El-Arbaein, Eg089 (NHMUK 2014.8.26.7), Eg091 (NHMUK 2014.8.26.8) from *D. dasyurus*, sampled on 21 August 2012 W. Gebel, Sinai Mountains, Egypt; all deposited at the Natural History Museum, London, UK.

*Vector*: currently unknown, but assumed to be a local species of ixodid tick.

*Representative sequences*: GenBank KJ908691 (18S rRNA gene); KJ908692 (ITS2 region); KM067276 (ITS1 region).

*Etymology*: The species is named for Professor Jerzy M. Behnke, the pioneer and the leader of studies on rodent parasites from isolated wadis in the Sinai Mountains of Egypt.

*ZooBank reference numbers:* pub: D3D8C6F4-796B-4E93-9DE4-CD6B7897E169

act: 7491E249-3966-4170-AC52-6D521D988672

Description

The organism is a typical small species of *Babesia*, with trophozoites occupying central to subcentral position within host erythrocytes (Figures 1B, E). On Giemsa stained slides, the cytoplasm is pale with a purple-staining nucleus around the periphery (Figure 1B, C). Trophozoites are mainly rounded, rarely slightly ovoid, less polymorphic than trophozoites of *B. microti* King’s 67 observed in BALB/c mice (Figure 1B–F). Trophozoite dimensions (diameter) of *B. behnkei* n. sp. were significantly smaller than those of *B. microti* King’s 67 [range 0.5-2.2 μm, mean ± SD 1.26 ± 0.35 μm (n = 212) *vs* range 0.6-3.0 μm, mean 1.46 ± 0.56 μm (n = 50); F_1,261_ *=* 8.48, *P =* 0.004, respectively]. Dividing forms, tetrads (resembling the Maltese cross) were observed and sometimes two forms in one red cell were recorded (Figure 1C).

### Field studies: ecology of *Babesia behnkei* n. sp.

Altogether, 1,021 rodents from the Sinai Mountains, Egypt, were sampled in four montane valleys (wadies) in 2000, 2004, 2008 and 2012, including 837 individuals of the spiny mouse *Acomys dimidiatus*, 73 *A. russatus* and 111 Wagner’s gerbils *Dipodillus dasyurus* (Table 1). Overall prevalence of *Babesia* spp. was the highest in Wagner’s gerbil (38.7%, Table 3) in comparison with *A. dimidiatus* or *A. russatus* (<10%, data not presented). Infections with *B. behnkei* were identified only in two isolated populations of *D. dasyurus*, from Wadi Gebel (66.1%) and from W. El-Arbaein (17.4%). Parasites were maintained in these populations over a period of at least 9 years, 2004-2012 (Table 3).

**Table 3:** **Prevalence of** ***B. behnkei*** **n. sp. in Wagner’s gerbils: no. of infected/examined hosts (prevalence in %)**

**Year of study**	**W. El-Arbaein**	**W. Gebel**	**W. Gharaba**	**W. Itlah**	**All sites**
2000	0/3 (0)*	0/6 (0)*	0/2 (0)*	0/2 (0)*	0/13 (0)*
2004	1/4 (25)	15/16 (93.8)	0/7 (0)	0/0	16/27 (59.3)
2008	1/2 (50)	6/15 (40)	0/2 (0)	0/0	7/19 (36.8)
2012	2/14 (14.3)	18/22 (81.8)	0/16 (0)	0/0	20/52 (38.5)
Total by site	4/23 (17.4)	39/59 (66.1)	0/27 (0)	0/2 (0)	43/111 (38.7)

*Prevalence only on the basis of microscopy; no DNA samples available for PCR.

### Genotyping and phylogenetic analysis

Thirty two isolates derived from *D. dasyurus* obtained over a 9 year period from two wadies (29 isolates from Wadi Gebel and 3 from Wadi El-Arbaein) (Table 2) were investigated by the analysis of near-full-length sequence of the 18S rRNA gene. All sequences were identical, indicating the presence of a single parasite species. A BLAST search in GenBank revealed no identical sequences in the database, therefore this new species was designated as *Babesia behnkei* n. sp. The highest homology (about 96%) found was with *B. lengau* from cheetahs [6] and with *B. vesperuginis* from bats *Pipistrellus* spp. in Cornwall, UK [53]. The 18S rRNA sequence for *Babesia behnkei* n. sp. differed from that for *B. lengau* by 43 nucleotides and from that for *B. microti* by 55 nucleotides (Additional file 1).

**Figure 2:**
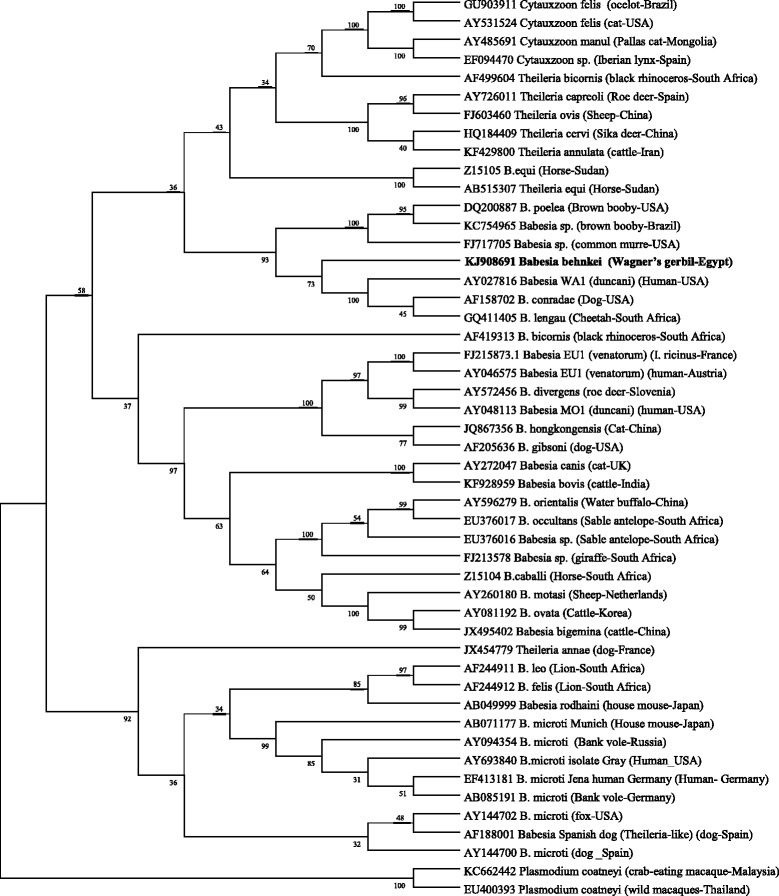
**Phylogenetic analysis of 18S rRNA sequences by the Maximum Likelihood method.** The evolutionary history was inferred based on the Tam3 (*I + G*) model. The tree with the highest log likelihood (0.0000) is shown. The percentages of replicate trees in which the associated taxa clustered together in the bootstrap test (1,000 replicates) are shown next to the branches [67]. The tree is drawn to scale, with branch lengths measured as the number of substitutions per site. The analysis involved 49 nucleotide sequences. All positions containing gaps and missing data were eliminated. Evolutionary analyses were conducted in MEGA v. 6.0 [51].

The phylogenetic analyses including sequences for *Babesia behnkei* n. sp. and for other species of *Babesia/Theileria* were conducted in MEGA v. 6.0 as detailed in the Methods section [51]. A representative tree for 18S rDNA sequences, obtained using the Maximum Likelihood method and a Tamura 3-parameter (I + G) model is presented in Figure 2. *Babesia behnkei* n. sp. clustered in a monophyletic group/clade with the African species *B. lengau* and with American zoonotic species *B. duncani* (*Babesia* WA1) and canine parasite *B. conradae* (‘Duncani group’[14]). This clade was distinct from *Babesia* spp. (*sensu stricto*), i.e. *B. bovis*, *B. canis*, *B. gibsoni*, *B. venatorum* [EU1] and *B. divergens*, as well as from the main *Theileria* spp. clade including *T. annulata*, and from the zoonotic and non-zoonotic *B. microti* strains (Figure 2).

**Figure 3:**
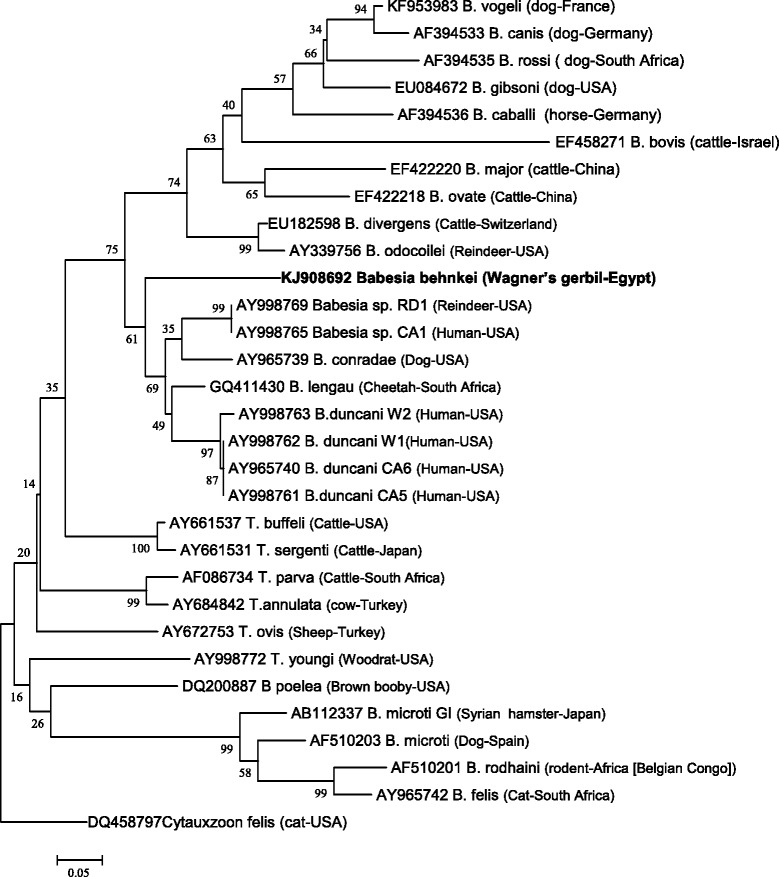
**Evolutionary relationships of the taxa based on ITS2 sequences.** The evolutionary history was inferred using the Neighbor-Joining method [68]. The optimal tree with the sum of branch length = 3.34829688 is shown. The percentages of replicate trees in which the associated taxa clustered together in the bootstrap test (1,000 replicates) are shown next to the branches [67]. The tree is drawn to scale, with branch lengths in the same units as those of the evolutionary distances used to infer the phylogenetic tree. The evolutionary distances were computed using the Kimura 2-parameter method [69] and are in the units of the number of base substitutions per site. The analysis involved 31 nucleotide sequences. All positions containing gaps and missing data were eliminated. There were a total of 107 positions in the final dataset. Evolutionary analyses were conducted in MEGA v. 6.0 [51].

Phylogenetic analysis of an approximately 315 bp region of ITS2 of four isolates by the neighbour-joining method with the Kimura two-parameter distance calculation revealed very similar results (Figure 3). *Babesia behnkei* n. sp. formed a monophyletic group with the American species and strains, *B. duncani* (*Babesia* WA1), *B. conradae* and others (‘Duncani group’[14]), and with the African *B. lengau.* Again, this clade was distant from *Babesia* spp. (*sensu stricto*), i.e. *B. divergens*, *B. major* and *B. gibsoni*, as well as from the main *Theileria* clade with *T. parva*, and from *B. microti* and related species (*B. rodhaini* and *B. felis*) (Figure 3).

Comparison of the ITS2 sequences for *Babesia behnkei* n. sp. with those for other species (*B. lengau*, *B. duncani* and *B. microti*) revealed low homology (Additional file 2). Similarly, the ITS1 sequence displayed low homology with a few known sequences for *Babesia* spp., including *B. microti* (Additional file 3).

## Discussion

Microscopic, molecular and phylogenetic analysis of the *Babesia* sp. infecting Wagner’s gerbil from the Sinai Mountains supported its differentiation from all known species and consequently the naming of a novel rodent species of piroplasms was justified. Infections with *Babesia behnkei* n. sp. were found in two isolated populations of *D. dasyurus* during a 9 year period (2004–2012). This novel species belongs to the ‘Duncani group’ (Clade VI) and is closely related to *B. lengau* and the human-infecting parasite *B. duncani* from North America.

Morphologically, *Babesia behnkei* n. sp. is indistinguishable from other small *Babesia* spp. but seems less polymorphic than *B. microti*. Dividing forms were observed rarely and parasitaemia exceeded 100 parasites per 200 fields of vision at × 1000 magnification in only 5 individuals. The majority of parasite trophozoites were regular and rounded.

Molecular and phylogenetic analysis of two widely used molecular markers (18S rDNA and ITS2) revealed that *Babesia behnkei* n. sp. is distinct from other known rodent *Babesia* spp. (*B. microti* and *B. rodhaini*), *Babesia* (*sensu stricto*) and *Theileria* spp. Analysis of both loci placed the new species in a recently distinguished ‘Duncani group’ (Clade VI [14]). This group is interesting because it consists of only a few named species and several unnamed piroplasms, including some pathogenic for humans [6,14]. Among the established species, there are two from North America, *B. duncani* (previously *Babesia* WA1), identified as an etiologic agent in human cases of babesiosis in western states of the USA [54], and *B. conradae*, described from a dog in California [55,56]. Among the parasites of the ‘Duncani group’, there is only one species from Africa, *B. lengau*, identified recently in cheetahs from South Africa [6]. However, another new strain/species of *Babesia* related to *B. lengau*, has been found recently in spotted hyenas from South Africa [57]; the latter still requires formal description. It is highly likely that this clade of piroplasms will be expanded in the future with new molecular studies on parasites from host species that have yet to be examined in Africa, America and elsewhere.

The pathogenicity of known and new *Babesia* species/strains differs extensively even among species from a single phylogenetic group. *Babesia lengau* appears to be nonpathogenic for cheetahs but is pathogenic for cats [6,12]. *Babesia conradae* causes haemolytic anaemia in dogs in California [55,56] and *B. duncani* may infect humans with an intact spleen or asplenic individuals, and infections in humans were reported to be subclinical or severe [17]. We have not observed any obvious symptoms of babesiosis in the Wagner’s gerbil (i.e. brown colored urine, chills, apathy).

Cases of human babesiosis have been recorded in Egypt [58-60] and interestingly, both North and South Sinai (our study site) governorates are considered to be endemic regions for babesiosis in Egypt [61]. The number of reported cases differs [33,61] and so far no molecular identification of the *Babesia* spp. involved in human cases has been carried out. The number of molecular studies on *Babesia* spp. infections in Egyptian ticks is also extremely limited and the results of the few published studies certainly need verification, i.e. the presence of *B. venatorum* (EU1) in ticks *Ixodes ricinus* or of *B. microti*, *B. venatorum* (EU1) and *B. bigemina* in rats/gerbils from Sinai Peninsula [62]. Because of the occurrence of human babesiosis in South Sinai, the high prevalence of *B. behnkei* n. sp. in a common rodent species from the region, the Wagner’s gerbil, and the close relationship between *B. behnkei* and the pathogenic *B. duncani*, the possibility of human infection with this novel species should be considered. Our as yet unpublished data indicate that the most common tick in the studied area is the camel tick, *Hyalomma dromedarii*, which also attaches to and feeds on humans. This tick species is certainly the main candidate for a possible vector of the new species of *Babesia*, especially because its juvenile stages were found feeding on rodents in Egypt [63]. Juvenile ticks were also collected from rodents in our study and we plan to screen these for the presence of the diagnostic marker for *B. behnkei* and hence to determine their role as a vector of *B. behnkei* in the region.

*Dipodillus (Gerbillus) dasyurus* was the second most numerous rodent species sampled throughout the 13 years of field work in Sinai. This solitary, burrowing species occurs in a variety of arid habitats, including desert, semi-desert and rocky habitats in hill country [64]. It is a common species, distributed mainly in the Nile Delta, the Sinai, Syria, Iraq and the Arabian Peninsula and it is listed as of Least Concern in the IUCN Red List of Threatened Species. Interestingly, we were able to amplify *Babesia* spp. DNA only from this host species, so it is likely that host specificity of *B. behnkei* n. sp. is high and that despite the concerns expressed above, it may not constitute a zoonotic treat to people in the region. The wide geographic range of *D. dasyurus* represents a particular challenge for the study of the distribution of the novel species of *Babesia*. On the other hand, the high rate of infection in gerbils registered in only two isolated wadis and the absence of the parasite throughout the period of study in other neighboring wadis, support the idea that *B. behnkei* might have evolved locally in these semi-isolated mountain populations of Wagner’s gerbils. In our studies on helminth communities in the same study sites, marked differences in community structure were noted between wadis [46]. Similarly, the prevalence of the intestinal protozoa and other haemoparasites (*Trypanosoma* spp., *Hepatozoon* spp.) differed markedly between rodent populations inhabiting these four sites [38]. Each wadi thus presents its own particular challenges for the animals that live there and local adaptation of parasites to their hosts and vice versa is to be expected [65,66]. In this particular case, *B. behnkei* n. sp. showed generally high prevalence (25–90%) in two wadis and was not detected at all in the other two of the four study sites monitored. Where present, it occurred in Wagner’s gerbils in each of the three surveys conducted over a period of 9 years (2004, 2008 and 2012). The Sinai Massif and its associated deep wadis constitute therefore an ideal location for studies of this type, testing the idea that parasites evolve and adapt locally to their hosts and assessing the role of gene flow and metapopulation structure for both hosts and parasites. In future work we hope to unravel further the intricacies of these relationships in the region, notably for haemoparasites such as *B. behnkei*.

## Conclusion

In conclusion, both ecological, phenotypic and phylogenetic analyses reported in this paper support the recognition of a new piroplasm, *B. behnkei* n. sp., infecting isolated populations of Wagner’s gerbil in Sinai as a distinct species.

## Additional files

Additional file 1:
**Alignment of 18S rRNA sequences.**
Additional file 2:
**Alignment of the ITS2 region.**
Additional file 3:
**Alignment of the ITS1 region.**
