# Supplementary material for: Babesia behnkei sp. nov., a novel Babesia species infecting isolated populations of Wagner’s gerbil, Dipodillus dasyurus, from the Sinai Mountains, Egypt
Source: Parasit Vectors. 2014 Dec 9;7:572. doi: 10.1186/s13071-014-0572-9 (PMC4271447; doi:10.1186/s13071-014-0572-9)
Supplement: Additional file 1: — Alignment of 18S rRNA sequences. [file 13071_2014_572_MOESM1_ESM.pdf]

```
                                     10         20         30         40         50         60         70         80         90        100
                             ....|....| ....|....| ....|....| ....|....| ....|....| ....|....| ....|....| ....|....| ....|....| ....|....|
AF244911_B._leo              ---------- ---------- -AGTCATATG CTTGTCTTAA AGATTAAGCC ATGCATGTCT TAGTATAAGC TTTTATACAG CGAAACTGCG AATGGCTCAT
EU376016_Babesia_sp._sable_ant ---------- ---------- ---------- ---------- ---------- ---------- ---------- ------ACGG TGAAACTGCG AATGGCTCAT
FJ213578_Babesia_sp._giraffe ---------- ---------- ---------- ---------- ---------- ---------- ---------- ------ACGG TGAAACTGCG AATGGCTCAT
AY027816_Babesia_WA1_USA     ----TGGTTG ATCCTGCCAG TAGTCATATG CTTGTCTTAA AGATTAAGCC ATGCATGTCT AAGTATAAAC TTTTATATGG TGAAACTGCG AATGGCTCAT
AY094354_B._microti_M._glareol ---------- ---------- ---------- ---------- ---------- ---------- ---------- ---------- ---------- ----------
AB071177_B._microti_Munich_M._ AACCTGGTTG ATCCTGCCAG TAGTCATATG CTTGTCTTAA AGATTAAGCC ATGCATGTCT TAGTATAAGC TTTTATACAG CGAAACTGCG AATGGCTCAT
AB085191_B._microti_M._glareol AACCTGGTTG ATCCTGCCAG TAGTCATATG CTTGTCTTAA AGATTAAGCC ATGCATGTCT TAGTATAAGC TTTTATACAG CGAAACTGCG AATGGCTCAT
AF499604_Theileria_bicornis  ---------- --------CA GTAGTCATAT GCTGTCTTAA AGATTAAGCC ATGCATGTCT AAGTATAAAC TTTTATATGG TGAAACTGCG AATGGCTCAT
AF419313_B._bicornis         ---------- ---------- -------ATG CTTGTCTTAA AGATTAAGCC ATGCATGTCT AAGTATAAGC TTCTATACGG TGAAACTGCG AATGGCTCAT
GQ411405_B._lengau           ---------- ---------- ---------- ---------- ---------- ---------- ---------C TTTTATATGG TAAAACTGCG AATGGCTCAT
AJ871610_Babesia_vesperuginis ---------- ---------- ---------- ---------- ---------- ---------- ---------- ---------- ---------- ----------
our_isolates                 ---------- ---------- ---------- ---------- ---------- ---------- ---------- ---------- ---------- ----------

                                    110        120        130        140        150        160        170        180        190        200
                             ....|....| ....|....| ....|....| ....|....| ....|....| ....|....| ....|....| ....|....| ....|....| ....|....|
AF244911_B._leo              TAAAACAGTT ATAGTTTATT TGATGTTCGT TTTA--CATG GATAACCGTG GTAATTCTAG GGCTAATACA TGCTCGAGGC GC--TTCG-- -GCGCGGCGT
EU376016_Babesia_sp._sable_ant TACAACAGTT ATAGTTTATT TGGTATTCGT TTT--CCATG GATAACCGTG CTAATTGTAG GGCTAATACA AGTTCGATGC -------TTT TTGGCGGCGT
FJ213578_Babesia_sp._giraffe TACAACAGTT ATAGTTTCTT TGGTATTCGT TTTTTCCATG GATAACCGTG CTAATTGTAG GGCTAATACA AGTTCGATGC C-----TTTT TTGGCGGCGT
AY027816_Babesia_WA1_USA     TACAACAGTT ATAGTTTATT TGAAAGTCGT TTTT-ACATG GATAACCGTG CTAATTGTAG GGCTAATACA TGCTCGAGGC CTTGGCTTCT GTCTTGGCTG
AY094354_B._microti_M._glareol ---------- ---------- ---------- ---------- ---------- ---------- ---------- ---------- ---------- ----------
AB071177_B._microti_Munich_M._ TAAAACAGTT ATAGTTTATT TGATGTTCGT TTTA--CATG GATAACCGTG GTAATTCTAG GGCTAATACA TGCTCGAGGC GC--GTTTTA CGCGTGGCGT
AB085191_B._microti_M._glareol TAAAACAGTT ATAGTTTATT TGATGTTCGT TTTA--CATG GATAACCGTG GTAATTCTAG GGCTAATACA TGCTCGAGGC GC--GTTTT- CGCGTGGCGT
AF499604_Theileria_bicornis  TACAACAGTT ATAGTTTATT TGATGTTCGT TTTT-ACATG GATAACCGTG CTAATTGTAG GGCTAATACA TGTTCGAGGG CT--GTTTTT CAGTCGGCGT
AF419313_B._bicornis         TACAACAGTT ATAGTTTACT TGACTTTGTT AATA--GATG GATAACCGTG CTAATTGTAG GGCTAATACA TGCATA---- ---------- ----TTGCGT
GQ411405_B._lengau           TAAAACAGTT ATAGTTTATT TGAAAGTCGT TTCT-ACATG GATAACCGTG CTAATTGTAG GGCTAATACA TGCTCGAGGT CATAGTTTCT ATGGCTGCGT
AJ871610_Babesia_vesperuginis ---------- ---------- ---------- ---------- ---------- ---------- ---------- ---------- ---------- ----------
our_isolates                 ---------- ---------- ---------- ---------- ---------- ---------- ---------- ---------- ---------- ----------

                                    210        220        230        240        250        260        270        280        290        300
                             ....|....| ....|....| ....|....| ....|....| ....|....| ....|....| ....|....| ....|....| ....|....| ....|....|
AF244911_B._leo              TTATTAGACT TAAACCAACC CCTTTGGGTA A-----CCGG TGATTCATAA TAAATCAGCG AATCGCATGG CTTT-GCCGG CGATGTATCA TTCAAGTTTC
EU376016_Babesia_sp._sable_ant TTATTAGCTT C--AAACCAA CCTCGGTTT- -------CGG TGATTCATAA TAAACTTGCG AATCGCATGC TTT---GCGG CGATGGCCCA TTCAAGTTTC
FJ213578_Babesia_sp._giraffe TTATTAGTTT TTTAAACCTC TCTTTTTTT- -------CGG TGATTCATAA TAAACTTGCG AATCGCATGC TTTT--GCGG CGATGGCCCA TTCAAGTTTC
AY027816_Babesia_WA1_USA     CGTTTATTAG ACTCGAAACC TTCCCGCTTG CGGTACTCGG TGATTCATAA TAAATTTGCG AATCGCATGG CTTTTGCCGG CGATGGTTCA TTCAAGTTTC
AY094354_B._microti_M._glareol ---------- ---------- ---------- ---------- ---------- ---------- ---------- ---------- ---------- ----------
AB071177_B._microti_Munich_M._ TTATTAGACT TTAACCAGCC CCTTTGGGTA A-----TCGG TGATTCATAA TAAATTAGCG AATCGCATGG CTTT-GCCGG CGATGTATCA TTCAAGTTTC
AB085191_B._microti_M._glareol TTATTAGACT TTAACCAACC C-TTTGGGTA A-----TCGG TGATTCATAA TAAATTAGCG AATCGCATGG CTTT-GCCGG CGATGTATCA TTCAAGTTTC
AF499604_Theileria_bicornis  TTATTAGTCT TTAAACCATC CCGCTTTTGC GGTTTTTTGG TGATTCATAA TAAACTTGCG AATCGCATGG CTTTTGCTGG CGATGTATCA TTCAAGTTTC
AF419313_B._bicornis         TTATTACCCT GAAACCACCT GCTTCGGCGG ACA---CTGG TGATTCATAA TAAAC-GGCT GATCGCGCA- --------AG CGACGTGTCA TTCAAGTTTC
GQ411405_B._lengau           TTATTAGACT CCAAACCTTC CCGCATTCTG CGGTTCTCGG TGATTCATAA TAAACTTGCG AATCGCATGG CTTT-GCCGG CGATGATTCA TTCAAGTTTC
AJ871610_Babesia_vesperuginis ---------- ---------- ---------- ---------- ---------- ---------- ---------- ---------- ---------- ----------
our_isolates                 ---------- ---------- ---------- ---------- ---------- ---------- ---------- ---------- ---------- ----------

                                    310        320        330        340        350        360        370        380        390        400
                             ....|....| ....|....| ....|....| ....|....| ....|....| ....|....| ....|....| ....|....| ....|....| ....|....|
AF244911_B._leo              TGACCTATCA GCTTTGGACG GTAGGGTATT GGCCTACCGG GGCGACGACG GGTGACGGGG AATTGGGGTT CGATTCCGGA GAGGGAGCCT GAGAAACGGC
EU376016_Babesia_sp._sable_ant TGACCCATCA GCTT--GACG GTAGGGTATT GGCCTACCGA GGCAGCAACG GGTAACGGGG AATTAGGGTT CGATTCCGGA GAGGGAGCCT GAGAAACGGC
FJ213578_Babesia_sp._giraffe TGACCCATCA GCTT--GACG GTAGGGTATT GGCCTACCGA GGCAGCAACG GGTAACGGGG AATTAGGGTT CGATTCCGGA GAGGGAGCCT GAGAAACGGC
AY027816_Babesia_WA1_USA     TGACCTATCA GCTTTGGACG GTAGGGTATT GGCCTACCGG GGCAGCGACG GGTAACGGGG AATTAGGGTT CGATTCCGGA GAGGGAGCCT GAGAAACGGC
AY094354_B._microti_M._glareol ---------- ---------- ---------- ---------- ---------- ---------- ---------- ---------- ---------- ----------
AB071177_B._microti_Munich_M._ TGACCTATCA GCTTTGGACG GTAGGGTATT GGCCTACCGG GGCGACGACG GGTGACGGGG AATTGGGGTT CGATTCCGGA GAGGGAGCCT GAGAAACGGC
AB085191_B._microti_M._glareol TGACCTATCA GCTTTGGACG GTAGGGTATT GGCCTACCGG GGCGACGACG GGTGACGGGG AATTGGGGTT CGATTCCGGA GAGGGAGCCT GAGAAACGGC
AF499604_Theileria_bicornis  TGACCTATCA GCTTTGGACG GTAGGGTATT GGCCTACCGG GGCAACGACG GGTAACGGGG AATTAGGGTT CGATTCCGGA GAGGGAGCCT GAGAAACGGC
AF419313_B._bicornis         TGACCTATCA GCTTTGGACG GTAGGGTATT GGCCTACCGG GGCAACGACG GGTAACGGGG AATTAGGGTT CGATTCCGGA GAGGGAGCCT GAGAAACGGC
GQ411405_B._lengau           TGACCTATCA GCTTTGGACG GTAGGGTATT GGCCTACCGG GGCAGCGACG GGTAACGGGG AATTAGGGTT CGATTCCGGA GAGGGAGCCT GAGAAACGGC
AJ871610_Babesia_vesperuginis ---------- ---------- ---------- ---------- ---------- ---------- ---------- ---------- ---------- ----------
our_isolates                 ---------- ---------- ---------- ---------- ---------- ---------- ---------- ---------- ---------- ----------

                                    410        420        430        440        450        460        470        480        490        500
                             ....|....| ....|....| ....|....| ....|....| ....|....| ....|....| ....|....| ....|....| ....|....| ....|....|
AF244911_B._leo              TACCACATCT AAGGAAGGCA GCAGGCGCGC AAATTACCCA ATCCTGACAC AGGGAGGTAG TGACAAGAAA TAACAATACA GGGCTTAT-A GTCTTGTAAT
EU376016_Babesia_sp._sable_ant TACCACATCT AAGGAAGGCA GCAGGCGCGC AAATTACCCA ATCCTGACAC AGGGAGGTAG TGACAAGAAA TAACAATACA GGGCTACT-- GTCTTGTAAT
FJ213578_Babesia_sp._giraffe TACCACATCT AAGGAAGGCA GCAGGCGCGC AAATTACCCA ATCCTGACAC AGGGAGGTAG TGACAAGAAA TAACAATACA GGGCTATT-- GTCTTGTAAT
AY027816_Babesia_WA1_USA     TACCACATCT AAGGAAGGCA GCAGGCGCGC AAATTACCCA ATACGGACAC CGTGAGGTAG TGACAAGAAA TAACAATACA GGGCTTTA-A GCTTTGTAAT
AY094354_B._microti_M._glareol ---------- ---------- --------CC ATATT-CCCA ATCCTGACAC AGGGAGGTAG TGACAAGAAA TAACAA-ACA GGGCTTAA-A GTCTTGTAAT
AB071177_B._microti_Munich_M._ TACCACATCT AAGGAAGGCA GCAGGCGCGC AAATTACCCA ATCCTGACAC AGGGAGGTAG TGACAAGAAA TAACAATACA GGGCTTAA-A GTCTTGTAAT
AB085191_B._microti_M._glareol TACCACATCT AAGGAAGGCA GCAGGCGCGC AAATTACCCA ATCCTGACAC AGGGAGGTAG TGACAAGAAA TAACAATACA GGGCTTAA-A GTCTTGTAAT
AF499604_Theileria_bicornis  TACCACATCT AAGGAAGGCA GCAGGCGCGC AAATTACCCA ATCCTGACAC AGGGAGGTAG TGACAAGAAA TAACAATACG GGGCTTAA-T GTCTTGTAAT
AF419313_B._bicornis         TACCACATCT AAGGAAGGCA GCAGGCGCGC AAATTACCCA ATCCTGAGAC AGGGAGGTAG TGACAAGAAA TAACAATACG GGGCTAAATA GTCCTGTAAT
GQ411405_B._lengau           TACCACATCT AAGGAAGGCA GCAGGCGCGC AAATTACCCA ATACGGACAC CGTGAGGTAG TGACAAGAAA TAACAATACA GGGCTTTA-A GCTTTGTAAT
AJ871610_Babesia_vesperuginis ---------- ---------A GCAGGCGCGC AAATTACCCA ATCCTGACAC AGGGAGGTAG TGACAAGAAA TAACAATACG GGGCTTAA-A GTCTTGTAAT
our_isolates                 ---------- ---------- ---------- ---------- ---------- ---------- ---------- ---------- ---------- ----------

                                    510        520        530        540        550        560        570        580        590        600
                             ....|....| ....|....| ....|....| ....|....| ....|....| ....|....| ....|....| ....|....| ....|....| ....|....|
AF244911_B._leo              TGGAATGATG GGAATCTAAA CCCTTCCCAG AGTATCAATT GGAGGGCAAG TCTGGTGCCA GCAGCCGCGG TAATTCCAGC TCCAATAGCG TATATTAAAG
EU376016_Babesia_sp._sable_ant TGGAATGATG GTGACTTAAA CCCTCACCAG AGTACCAATT GGAGGGCAAG TCTGGTGCCA GCAGCCGCGG TAATTCCAGC TCCAATAGCG TATATTAAAC
FJ213578_Babesia_sp._giraffe TGGAATGATG GTGACTTAAA CCCTCACCAG AGTACCAATT GGAGGGCAAG TCTGGTGCCA GCAGCCGCGG TAATTCCAGC TCCAATAGCG TATATTAAAC
AY027816_Babesia_WA1_USA     TGGAATGATG GGAATCCAAA CCCCTTCCAG AGTATCAATT GGAGGGCAAG TCTGGTGCCA GCAGCCGCGG TAATTCCAGC TCCAATAGCG TATATTAAAC
AY094354_B._microti_M._glareol TGGAATGATG GGAATCTAAA CCCTTCCCAG AGTATCAATT GGAGGGCAAG TCTGGTGCCA GCAGCCGCGG TAATTCCAGC TCCAATAGCG TATATTAAAG
AB071177_B._microti_Munich_M._ TGGAATGATG GGAATCTAAA CCCTTCCCAG AGTATCAATT GGAGGGCAAG TCTGGTGCCA GCAGCCGCGG TAATTCCAGC TCCAATAGCG TATATTAAAG
AB085191_B._microti_M._glareol TGGAATGATG GGAATCTAAA CCCTTCCCAG AGTATCAATT GGAGGGCAAG TCTGGTGCCA GCAGCCGCGG TAATTCCAGC TCCAATAGCG TATATTAAAG
AF499604_Theileria_bicornis  TGGAATGATG GGAATTTAAA CCCCTTCCAG AGTATCAATT GGAGGGCAAG TCTGGTGCCA GCAGCCGCGG TAATTCCAGC TCCAATAGCG TATATTAAAC
AF419313_B._bicornis         TGGAATGATG GGAACTTACA GGCGTTCCAG AGTATCAATT GGAGGGCAAG TCTGGTGCCA GCAGCCGCGG TAATTCCAGC TCCAATAGCG TATATTAAAC
GQ411405_B._lengau           TGGAATGATG GGAATTTAAA CCCCTTCCAG AGTATCAATT GGAGGGCAAG TCTGGTGCCA GCAGCCGCGG TAATTCCAGC TCCAATAGCG TATATTAAAC
AJ871610_Babesia_vesperuginis TGGAATGATG GGAATCTAAA CCCCTTCCAG AGTATCAATT GGAGGGCAAG TCTGGTGCCA GCAGCCGCGG TAATTCCAGC TCCAATAGCG TATATTAAAC
our_isolates                 ---------- ---------- ---------- ---------- ---------- ---------- ---------- ---------- ---------- ----------

                                    610        620        630        640        650        660        670        680        690        700
                             ....|....| ....|....| ....|....| ....|....| ....|....| ....|....| ....|....| ....|....| ....|....| ....|....|
AF244911_B._leo              TTGTTGCAGT TAAGAAGCTC GTAGTTGAAT TTCTGCCTTG TCTTT-GGAC TCGCTTCCAA .C....TCCA TTC.ACT... .A.CT.T... .A.CT.G...
EU376016_Babesia_sp._sable_ant TTGTTGCAGT TAAAAAGCTC GTAGTTGAA- ---------- ---------- -----T...A .C------T. .TA.C.T..A ....G.---. TC.TTAGC.A
FJ213578_Babesia_sp._giraffe TTGTTGCAGT TAAAAAGCTC GTAGTTGAA- ---------- ---------- -----T...A .C...--.T. TTC..CT.CC ....T.C.G. ...TCG.C.A
AY027816_Babesia_WA1_USA     TTGTTGCAGT TAAAAAGCTC GTAGTTGAAC TTCTGCCGCT TTGGCCTTTC GTTCC.C.T. ........T. CGCC...... ...A.CT... .CGGTGG..-
AY094354_B._microti_M._glareol TTGTTGCAGT TAAGAAGCTC GTAGTTGAAT TTCTGCCTTG TCATT-AATC TCGCTTCCGA .C....TTT. .TT.ACT... .A.CT.-... .A.TTGG.GC
AB071177_B._microti_Munich_M._ TTGTTGCAGT TAAGAAGCTC GTAGTTGAAT TTCTGCCTTG TCATT-AATC TCGCTT.CGA .C...ATTT. .TT.ACT... .A.CT.-... .A.TTGG.AT
AB085191_B._microti_M._glareol TTGTTGCAGT TAAGAAGCTC GTAGTTGAAT TTCTGCCTTG TCATT-AATC TCGCTTCCGA .C....TTT. .TT.GCT... .A.CT.-... .A.TTGG.GC
AF499604_Theileria_bicornis  TTGTTGCAGT TAAAAAGCTC GTAGTTGAAT TTCTGCTGCT TCGCT--TTC GTTCCT..TT ..-.....T. TGC..T.... ....T.T... ...G.-....
AF419313_B._bicornis         TTGTTGCAGT TAAAAAGCTC GTAGTTGAAT TTCTGCTGCG TCGGTTGGTG GTTCCT..T. .TAAA...CC TT...C.... T...T.-.C. ...GCC....
GQ411405_B._lengau           TTGTTGCAGT TAAAAAGCTC GTAGTTGAAC TTCTGCTGTC TGGA--TTTC GTTCC...T. ........C. CTCC..A.A. .A....T.C. ........G.
AJ871610_Babesia_vesperuginis TTGTTGCAGT TAAAAAGCTC GTAGTTGAAC TTCTGCTGTC AGGTT--TTC GTTCCT.... ........TC ..CC...... ....T.T... .G.G.A...A
our_isolates                 ---------- ---------- ---------- ---------- ---------- -----CTTCG GGGTTTCGAT AAGGTGGTGG CTTTCT-CTG GTTAATTTTG

                                    710        720        730        740        750        760        770        780        790        800
                             ....|....| ....|....| ....|....| ....|....| ....|....| ....|....| ....|....| ....|....| ....|....| ....|....|
AF244911_B._leo              CT.------- G..G...... ....-.T... T......... ...C...... .T....A... ...CA..C.. .......... A......... ..........
EU376016_Babesia_sp._sable_ant TT.------- -G--.....G TT.-----.. -......... .......... .T........ ...-.....T .......... .G........ .....GG...
FJ213578_Babesia_sp._giraffe CT.------- -.GCT..... TT.-----.. -......... .......... .T........ ...-.....T .......... .G........ ......G...
```

```
AY027816_Babesia_WA1_USA       ---------- -.TC.A...G ....-.-... -......... .......... .T........ ...-...... .......... .......... ..........
AY094354_B._microti_M._glareol CT.C----.. GT.CTA.... ....-G.... -......... ...C...... .T.....A.. ...-A..C.. .......... A......... .....G....
AB071177_B._microti_Munich_M._ C...AATT.. TT.CTA.C.. ....-..... -......... ...C...... .T.....A.. ...CA..... .......... A......... .....G....
AB085191_B._microti_M._glareol CT.C----.. GT.CTA.... ....-G.... -......... ...C...... .T.....A.. ...-A..C.. .......... A......... .....G....
AF499604_Theileria_bicornis    G...----.. G.C.T..... ....A.T... T......... .......... .......... ...-...... .......... .T........ ..........
AF419313_B._bicornis           ---------- -----GG... ..G.-A..G. T......... .......... ......G... ...-..G... .......... CG........ ....CGG...
GQ411405_B._lengau             G...---... T.C....... ..G.-.-... -......... .......... .T........ ...-...... .......... .......... ..........
AJ871610_Babesia_vesperuginis  G...----C. G.T.AA...C ....-..... T......... .......... .......... ...-...... .G........ .......... ..........
our_isolates                   -CTT---CGG -CATCTTTTT CCAG-TATTT -ACTTTGAGA AAATTAGAGT GCTTCAAGCA GGC-TTTTGC CTTGAATACT TCAGCATGGA ATAATAAAGT

                                      810        820        830        840        850        860        870        880        890        900
                               ....|....| ....|....| ....|....| ....|....| ....|....| ....|....| ....|....| ....|....| ....|....| ....|....|
AF244911_B._leo                .......... .......... ......---- .TC.-A.... T......... ........G. .......... .......... .......... ..........
EU376016_Babesia_sp._sable_ant .....C.... .......... ......---- .TG--T...T GG........ .......... G......... .......... .......... ..........
FJ213578_Babesia_sp._giraffe   .....C.... ....TC.... ......---- .T..TT...C TG........ .......G.. G......... .......... .......... ..........
AY027816_Babesia_WA1_USA       .......... .......... ......---- ..A.-..... .......... .......... .......... .......... .......... ..........
AY094354_B._microti_M._glareol .......... .......... ......---- AT..-AG... G......... ........G. .......... .......... .......... ..........
AB071177_B._microti_Munich_M._ .......... .......... ......---- AT..-AG... G......... ........G. .......... .......... .......... ..........
AB085191_B._microti_M._glareol .......... .......... ......---- AT..-AG... G......... ........G. .......... .......... .......... ..........
AF499604_Theileria_bicornis    ........T. G.....A... ......GGTT .T..A..... .......... .......... .......... .......... .......... ..........
AF419313_B._bicornis           .......... .......... ......---- -TGAGAG... G......... .......... .......... .......... G..A...... ..........
GQ411405_B._lengau             .......... .......... ......---- ..A.-AG... .......... .......... .......... .......... .......... ..........
AJ871610_Babesia_vesperuginis  .......... .......... ......---- ....A-.... .......... .......... .......... .......... .......... ..........
our_isolates                   AGGACTTTGG TTCTATTTTG TTGGTT---- TCTG-GACCA AAGTAATGGT TAATAGGAAC AGTTGGGGGC ATTCGTATTT AACTGTCAGA GGTGAAATTC

                                      910        920        930        940        950        960        970        980        990       1000
                               ....|....| ....|....| ....|....| ....|....| ....|....| ....|....| ....|....| ....|....| ....|....| ....|....|
AF244911_B._leo                .......... .......... .......... .......... .......... .......... .......... .......... .......... ..........
EU376016_Babesia_sp._sable_ant .......... .......... .......... .......... .....C.... C......... .......... .......... .......... ..........
FJ213578_Babesia_sp._giraffe   .......... .......... .......... .......... .....C.... C......... .......... .......... .......... ..........
AY027816_Babesia_WA1_USA       .......... .......... .......... .......... .......... .......... .......... .......C.. .......... ..........
AY094354_B._microti_M._glareol .......... .......... .......... .......... .......... .......... .......... .......... .......... ..........
AB071177_B._microti_Munich_M._ .......... .......... .......... .......... .......... .......... .......... .......... .......... ..........
AB085191_B._microti_M._glareol .......... .......... .......... .......... .......... .......... .......... .......... .......... ..........
AF499604_Theileria_bicornis    .......... .......... .......... .......... .......... .......... .......... .......... .......... ..........
AF419313_B._bicornis           .......... C........G .......... .......... .......... .......... .......... .......... .......... ..........
GQ411405_B._lengau             .......... .......... .......... .......... .......... .......... .......... .......C.. .......... ..........
AJ871610_Babesia_vesperuginis  .......... .......... .......... .......... .......... .......... .......... .......... .......... ..........
our_isolates                   TTAGATTTGT TAAAGACGAA CTACTGCGAA AGCATTTGCC AAGGATGTTT TCATTAATCA AGAACGAAAG TTAGGGGATC GAAGACGATC AGATACCGTC

                                     1010       1020       1030       1040       1050       1060       1070       1080       1090       1100
                               ....|....| ....|....| ....|....| ....|....| ....|....| ....|....| ....|....| ....|....| ....|....| ....|....|
AF244911_B._leo                .......... ..A....... .......... .......... ......G... .T.-...... .......... .......... .......... ..........
EU376016_Babesia_sp._sable_ant .......... ..A....... .......... G......... .......... CC---..... .......... .......... .......... .A........
FJ213578_Babesia_sp._giraffe   .......... ..A....... .......... G......... C......... TCTT...... .......... .......... .......... ..........
AY027816_Babesia_WA1_USA       .......... .TA....... .......... .......... .......... ...-...... .......... .......... .......... ..........
AY094354_B._microti_M._glareol .......... ..A....... .......... .......... ......G... ...-...... .......... .......... .......... ..........
AB071177_B._microti_Munich_M._ .......... ..A....... .......... .......... ......G... ...-...... .......... .......... .......... ..........
AB085191_B._microti_M._glareol .......... ..A....... .......... .......... ......G... ...-...... .......... .......... .......... ..........
AF499604_Theileria_bicornis    .......... ..A....... .......... .......... ......G... ...-...... .......... .......... .......... ..........
AF419313_B._bicornis           .......... ........G. .......... ....C..... .....---.. .T.--..... ....G.G... .......... .......... ..........
GQ411405_B._lengau             .......... .TA....... .......... .......... .......... ...-...... .......... .......... .......... ..........
AJ871610_Babesia_vesperuginis  .......... ..A....... .......... .......... .........A ...-....-- ---------- ---------- ---------- ----------
our_isolates                   GTAGTCCTAA CCGTAAACTA TGCCGACTAG AGATTGGAGG TCGTCATTTT AAA-CGACTC CTTCAGCACC TTGAGAGAAA TCAAAGTCTT TGGGTTCTGG

                                     1110       1120       1130       1140       1150       1160       1170       1180       1190       1200
                               ....|....| ....|....| ....|....| ....|....| ....|....| ....|....| ....|....| ....|....| ....|....| ....|....|
AF244911_B._leo                .......... ........T. .......... .......... .......... .......... .......... .......... .......... .....C....
EU376016_Babesia_sp._sable_ant .......... ........T. .......... .......... .......... .......... .......... .......... .......... ..G..C....
FJ213578_Babesia_sp._giraffe   .......... ........T. .......... .......... .......... .......... .......... .......... .......... ..G..C....
AY027816_Babesia_WA1_USA       .......... .......... .......... .......... .......... .......... .......... .......... .......... ..G..C....
AY094354_B._microti_M._glareol .......... ........T. .......... .......... .......... .......... .......... .......... .......... .....C....
AB071177_B._microti_Munich_M._ .......... ........T. .......... .......... .......... .......... .......... .......... .......... .....C....
AB085191_B._microti_M._glareol .......... ........T. .......... .......... .......... .......... .......... .......... .......... .....C....
AF499604_Theileria_bicornis    .......... .......... .......... .......... .......... .......... .......... .......... .......... ..........
AF419313_B._bicornis           .......... .......... .......... .......... .......... .......... .......... .......... .......... ..G..G....
GQ411405_B._lengau             .......... .......... .......... .......... .......... .......... .......... .......... .......... ..G..C....
AJ871610_Babesia_vesperuginis  ---------- ---------- ---------- ---------- ---------- ---------- ---------- ---------- ---------- ----------
our_isolates                   GGGGAGTATG GTCGCAAGGC TGAAACTTAA AGGAATTGAC GGAAGGGCAC CACCAGGCGT GGAGCCTGCG GCTTAATTTG ACTCAACACG GGAAAACTCA

                                     1210       1220       1230       1240       1250       1260       1270       1280       1290       1300
                               ....|....| ....|....| ....|....| ....|....| ....|....| ....|....| ....|....| ....|....| ....|....| ....|....|
AF244911_B._leo                .......... ...T..AG.T .......... .......... .......... ..A....... .......... .......... .......... ..........
EU376016_Babesia_sp._sable_ant .......... ...AG.A... .......... .......... .......... ........A. .......... .......... .......... ..........
FJ213578_Babesia_sp._giraffe   .......... ...A..TT.. .......... .......... .......... ........A. .......... .......... .......... ....C.....
AY027816_Babesia_WA1_USA       .......... ...T..TT.. .......... .......... .......... ..A.....A. .......... .......... .......... ..........
AY094354_B._microti_M._glareol .......... ...T..AG.. .......... .......... .......... ..A....... .......... .......... .......... ..........
AB071177_B._microti_Munich_M._ .......... ...T..AG.. .......... .......... .......... ..A....... .......... .......... .......... ..........
AB085191_B._microti_M._glareol .......... ...T..AG.. .......... .......... .......... ..A....... .......... .......... .......... ..........
AF499604_Theileria_bicornis    .......... ...A...... .......... .......... .......... .......... .......... .......... .......... ..........
AF419313_B._bicornis           .......GG. ...A...... .......... .......... .......... .......... .......... .......... .......... ..........
GQ411405_B._lengau             .......... ...T..TT.. .......... .......... .......... ..A.....A. .......... .......... .......... ..........
AJ871610_Babesia_vesperuginis  ---------- ---------- ---------- ---------- ---------- ---------- ---------- ---------- ---------- ----------
our_isolates                   CCAGGTCCAG ACAGAGGAAG GATTGACAGA TTGATAGCTC TTTCTTGATT CTTTGGGTGG TGGTGCATGG CCGTTCTTAG TTGGTGGAGT GATTTGTCTG

                                     1310       1320       1330       1340       1350       1360       1370       1380       1390       1400
                               ....|....| ....|....| ....|....| ....|....| ....|....| ....|....| ....|....| ....|....| ....|....| ....|....|
AF244911_B._leo                .......... .......... .......... .........T AGGATACGAG ....G.CC.- .C......GT .A.C...... .......... .....T....
EU376016_Babesia_sp._sable_ant .......... .......... .......... ......TT.. .---..--AG C.CG...C-- --G..TG-T. ..A....... .......... C.....CTTC
FJ213578_Babesia_sp._giraffe   .......... .......... .......... ......TT.. .---..TT.G T..CTT..-- --G..T..TT ..AA...... .......... C.....CTTC
AY027816_Babesia_WA1_USA       .......... .......... .......... .......... .C........ ....T..C-- -....T.... .......... .......... ..........
AY094354_B._microti_M._glareol .......... .......... .......... .........T AGGATCTG.G .C..G....- .C...TC... .A.C...... .......... .....T....
AB071177_B._microti_Munich_M._ .......... .......... .......... .........T AGGATCTG.G .C..G..A.- .C...TC... .A.C...... .......... .....T....
AB085191_B._microti_M._glareol .......... .......... .......... .........T AGGATCTG.G .C..G....- .C...TC... .A.C...... .......... .....T....
AF499604_Theileria_bicornis    .......... .......... .......... .......... .G.TT..... .......... .........A .A.C...... .......... ..........
AF419313_B._bicornis           .......... .......... .......G.. .......... .C..TC.G.. ...GGG.AA- -CC..TC.GA CG.-...... .......... ..........
GQ411405_B._lengau             .......... .......... .......... .......... .C........ ........-- .....T.... .C........ .......... ..........
AJ871610_Babesia_vesperuginis  ---------- ---------- ---------- ---------- ---------- ---------- ---------- ---------- ---------- ----------
our_isolates                   GTTAATTCCG TTAACGAACG AGACCTTAAC CTGCTAAATA GTAGCTGAGA ATAAACTTTT GTTGTCTCAG TTTTGCTTCT TAGAGGGACT TTGCGGTCAT

                                     1410       1420       1430       1440       1450       1460       1470       1480       1490       1500
                               ....|....| ....|....| ....|....| ....|....| ....|....| ....|....| ....|....| ....|....| ....|....| ....|....|
AF244911_B._leo                ...A...... .......... .......... .......... .......... .......... .......... .......... .....A.... .-..T-....
EU376016_Babesia_sp._sable_ant ..G.....G. .....T.... .......... .......... .......... .G........ .......... .......... .AG....... .-...T...G
FJ213578_Babesia_sp._giraffe   ..G.....G. .....T.... .......... .......... .......... .......... .......... .......... ..G....T.. .-...T...G
AY027816_Babesia_WA1_USA       .......... .....T.... .......... .......... .......... .......... .......... .......... .......... .T...T....
AY094354_B._microti_M._glareol ...A...... .......... .......... .......... .......... .......... .......... .......... .....A.... .-..TT....
AB071177_B._microti_Munich_M._ ...A...... .......... .......... .......... .......... .......... .......... .......... .....A.... .-..TT....
AB085191_B._microti_M._glareol ...A...... .......... .......... .......... .......... .......... .......... .......... .....A.... .-..TT....
AF499604_Theileria_bicornis    .......... .....T.... .......... .......... .......... .......... .......... .......... .......... .-...A....
```

```
AF419313_B._bicornis           .......... .....T.G.. .......... .......... .......... .......... .......... .......... ...T.A.... .-G..T....
GQ411405_B._lengau             .......... .....T.... .......... .......... .......... .......... .......... .......... .......... .T...T....
AJ871610_Babesia_vesperuginis  ---------- ---------- ---------- ---------- ---------- ---------- ---------- ---------- ---------- ----------
our_isolates                   AAATCGCAAG GAAGTGTAAG GCAATAACAG GTCTGTGATG CCCTTAGATG TCCTGGGCTG CACGCGCGCT ACACTGATGC ATTCATCGAG TGTTACCCTT

                                        1510       1520       1530       1540       1550       1560       1570       1580       1590       1600
                               ....|....| ....|....| ....|....| ....|....| ....|....| ....|....| ....|....| ....|....| ....|....| ....|....|
AF244911_B._leo                .G...G.... TCCG.....T ...AC..... ........A. ......A... .......... .......T.. ...G...... .......... ..........
EU376016_Babesia_sp._sable_ant CT........ CGCG.....C .AC-....C. ........TC .......... .T........ C.A....... ...G...... ........T. ...A......
FJ213578_Babesia_sp._giraffe   CT........ CG.G.....T .A.-....C. ........TC .......... .T........ C.A....... ...G...... ........T. ...A......
AY027816_Babesia_WA1_USA       .......... ...T.....T ...-...... ........A. .......... .......... .......... ...G...... .......... ..........
AY094354_B._microti_M._glareol .G...TCG.. TCCG.....T ...AC..... ........A. ......A... .......... .......T.. ...G...... .......... ..........
AB071177_B._microti_Munich_M._ .G...T.G.. TCCG.....T ...AC..... ........A. ......A... .......... .......T.. ...G...... .......... ..........
AB085191_B._microti_M._glareol .G...TCG.. TCCG.....T ...AC..... ........A. ......A... .......... .......T.. ...G...... .......... ..........
AF499604_Theileria_bicornis    .G....G... C..G.....T ...-...... ........AC .......... .......... .......... ...G...... .......... ..........
AF419313_B._bicornis           .G....G... TC.G.....T ...-G..... ........AC .........C C....T.... C..GG..... ...G...... .......... ..........
GQ411405_B._lengau             .......... ...T.....T ...-...... ........A. .......... .......... .......... ...G...... .......... ..........
AJ871610_Babesia_vesperuginis  ---------- ---------- ---------- ---------- ---------- ---------- ---------- ---------- ---------- ----------
our_isolates                   GCCCGAAAGG GTTAGGTAAA CTT-TAGTAT GCATCGTGGT GGGGATTGAT TATTGCAATT ATTAATCATG AACTAGGAAT GCCTAGTAGG CGCGAGTCAT

                                        1610       1620       1630       1640       1650       1660       1670       1680       1690       1700
                               ....|....| ....|....| ....|....| ....|....| ....|....| ....|....| ....|....| ....|....| ....|....| ....|....|
AF244911_B._leo                ........GC CGACTACGTC CCTGCCCTTT GTACACACCG CCCGTC--GC TCCTACCGAT CGAGTGATCC GGTGAATTAT TCGGACCGGA AAACTTA-GA
EU376016_Babesia_sp._sable_ant .....T..GC AGATTACGTC CCTGCCCTTT GTACACACCG CCCGTC--GC TCCTACCGAT CGAGTGATCC GGTGAATTAT TCGGACCGTG GCTTTTCCGA
FJ213578_Babesia_sp._giraffe   .....T..GC AGATTACGTC CCTGCCCTTT GTACACACCG CCCGTC--GC TCCTACCGAT CGAGTGATCC GGTGAATTAT TCGGACCGTG GCTTTTCCGA
AY027816_Babesia_WA1_USA       ........GC CGACTACGTC CCTGCCCTTT GTACACACCG CCCGTC--GC TCCTACCGAT CGAGTGATCC GGTGAATTAT TCGGACCGTG ACGCTTCTAA
AY094354_B._microti_M._glareol ........GC CGACTACGTC CCTGCCCTTT GTACACACCG CCCGTC--GC TCCTACCGAT CGAGTGATCC GGTGAATTAT TCGGACCAAG AAACGTG-GA
AB071177_B._microti_Munich_M._ ........GC CGACTACGTC CCTGCCCTTT GTACACACCG CCCGTC--GC TCCTACCGAT CGAGTGATCC GGTGAATTAT TCGGACTGAG AAACGTG-GA
AB085191_B._microti_M._glareol ........GC CGACTACGTC CCTGCCCTTT GTACACACCG CCCGTC--GC TCCTACCGAT CGAGTGATCC GGTGAATTAT TCGGACCAAG AAACGTG-GA
AF499604_Theileria_bicornis    ........GC CGACTACGTC CCTGCCCTTT GTACACACCG CCCCGTCAGC TCCTACCGAT CGAGTGATCC GGTGAATTAT TCGGACCGTG ATGTTTCTAT
AF419313_B._bicornis           ........GC CGATTACGTC CCTGCCCTTT GTACACACCG CCCGTC--GC TCCTACCGAT CGAGTGATCC GGTGAATTAT TCGGACCGTG GTTTTTGTAG
GQ411405_B._lengau             ........GC CGACTACGTC CCTGCCCTTT GTACACACCG CCCGTC--GC TCCTACCGAT CGAGTGATCC GGTGAATTAT CTCGGACCGT GACGTTTCTA
AJ871610_Babesia_vesperuginis  ---------- ---------- ---------- ---------- ---------- ---------- ---------- ---------- ---------- ----------
our_isolates                   CAGCTCGT-- ---------- ---------- ---------- ---------- ---------- ---------- ---------- ---------- ----------

                                        1710       1720       1730       1740       1750       1760       1770       1780       1790       1800
                               ....|....| ....|....| ....|....| ....|....| ....|....| ....|....| ....|....| ....|....| ....|....| ....|....|
AF244911_B._leo                ----TTCGTC TAAAGTTTTT TGGGAAGTTT TGTGAACCTT ATCACTTAAA ---------- ---------- ---------- ---------- ----------
EU376016_Babesia_sp._sable_ant ----TTCGTC GGTTTCGCCT AGGGAAGTTT TG-------- ---------- ---------- ---------- ---------- ---------- ----------
FJ213578_Babesia_sp._giraffe   ----TTCGTC GGTTTTGCCT AGGGAAGTTT TG-------- ---------- ---------- ---------- ---------- ---------- ----------
AY027816_Babesia_WA1_USA       ----TTCGTT AGAAATGTCT AGGGAAGTTT TGTGAACCTT ATCACTTAAA GGAAGGAGAA GTCCGTAACA AGGTTTCC-- ---------- ----------
AY094354_B._microti_M._glareol ----TTCGTC CTTCGTTTTT TGGAAAGTTT TGTGAACCTT ATCACTTAAA GGAAGGAGAA GTCGTAACAA GGTTTCCGTA GGTGAACCTG CAGAAAGGAA
AB071177_B._microti_Munich_M._ ----TTCGTC CTTCGTTTTT CGGAAAGTTT TGTGAACCTT ATCACTTAAA GGAAGGAGAA GTCGTAACAA GGTTTCCGTA GGTGAACCTG CAGAAGGAT-
AB085191_B._microti_M._glareol ----TTCGTC CTTCGTTTTT TGGAAAGTTT TGTGAACCTT ATCACTTAAA GGAAGGAG-- ---------- ---------- ---------- ----------
AF499604_Theileria_bicornis    ----TTTTTA GAAACGTCTA GGGAAGTTTT G--------- ---------- ---------- ---------- ---------- ---------- ----------
AF419313_B._bicornis           GGCTTGCTCT ACTATTACCT AGGGAAGTTT TGTGAACCTT ATCACTTAAA GGAAGGAGAA GT-------- ---------- ---------- ----------
GQ411405_B._lengau             ---------- ---------- ---------- ---------- ---------- ---------- ---------- ---------- ---------- ----------
AJ871610_Babesia_vesperuginis  ---------- ---------- ---------- ---------- ---------- ---------- ---------- ---------- ---------- ----------
our_isolates                   ---------- ---------- ---------- ---------- ---------- ---------- ---------- ---------- ---------- ----------


                               ....|....
AF244911_B._leo                ---------
EU376016_Babesia_sp._sable_ant ---------
FJ213578_Babesia_sp._giraffe   ---------
AY027816_Babesia_WA1_USA       ---------
AY094354_B._microti_M._glareol TCAAGCTTT
AB071177_B._microti_Munich_M._ ---------
AB085191_B._microti_M._glareol ---------
AF499604_Theileria_bicornis    ---------
AF419313_B._bicornis           ---------
GQ411405_B._lengau             ---------
AJ871610_Babesia_vesperuginis  ---------
our_isolates                   ---------
```
