# Supplementary material for: Babesia behnkei sp. nov., a novel Babesia species infecting isolated populations of Wagner’s gerbil, Dipodillus dasyurus, from the Sinai Mountains, Egypt
Source: Parasit Vectors. 2014 Dec 9;7:572. doi: 10.1186/s13071-014-0572-9 (PMC4271447; doi:10.1186/s13071-014-0572-9)
Supplement: Additional file 2: — Alignment of the ITS2 region. [file 13071_2014_572_MOESM2_ESM.pdf]

```
                                     10         20         30         40         50         60         70         80         90        100
                             ....|....| ....|....| ....|....| ....|....| ....|....| ....|....| ....|....| ....|....| ....|....| ....|....|
GQ411430_B._lengau           ---------- ---------- ---------- ---------- ---------- --TGTGAATT GCACACTCCT GCGAAT-CAA TAGACTTCTG AACGTATCAT
our_sequences                ---------- ---------- ---------- ---------- ---------- --.....-.. ..GA...T.. ......G... ..A.TC.... ..........
AY965739_B._conradae         ---------- ---------- ---------- ----GCGAAT TGCGATAACC AT........ ....G..... ......-... C....C.... ..........
AY998763_B.duncani_W2        ---------- ---------- ---------- -----CGAAT TGCGATAATC AT........ ....G..... ......-... .......... ..........
AY998762_B._duncani_W1       GGATGTCTTG GCTCACACAA CGATGAAGGA CGCAGCGAAT TGCGATAATC AT........ ....G..... ......-... .......... ..........
AY998769_Babesia_sp._RD1     GGATGTCTTG GCTCACACAA CGATGAAGGA CGCAGCGAAT TGCGATACGC AT........ ...AG..... ......-... C....C.... ..........
AY998765_Babesia_sp._CA1     ---------- ---------- ---------- ----GCGAAT TGCGATACGC AT........ ...AG..... ......-... C....C.... ..........
AY965740_B._duncani_CA6      GGATGTCTTG GCTCACACAA CGATGAAGGA CGCAGCGAAT TGCGATAATC AT........ ....G..... ......-... .......... ..........
AY998761_B.duncani_CA5       ---------- ---------- ---------- ---------- -------ATC AT........ ....G..... ......-... .......... ..........
AB112337_B._microti_GI       GGATGTCTCG GCTCACACAA CGATGAAGGA CGCAGCGAAG TGCGATAATC AT........ ...G.A.TTA ..A...-... C..GT..... ..T....TG.
AF510203_B._microti          GGATGTCTCG GCTCGCACAA CGATGAAGGA CGCAGCGAAT TGCGATAATC AT........ ...G.A.TTA ..A...-... C...T..... ..T....TG.

                                    110        120        130        140        150        160        170        180        190        200
                             ....|....| ....|....| ....|....| ....|....| ....|....| ....|....| ....|....| ....|....| ....|....| ....|....|
GQ411430_B._lengau           ACACACCGCC TCTGCTTG-C ATGCGGTACT CCCATTTCAG TGAACTTTAA A-TCCTAAAA ACAAACCACT TTTGGTGGT- -------TAT ----------
our_sequences                .......... ......C.-. .......... ...G...... ....TCA... TA........ G...-.AC.. ...------- ---------- ----------
AY965739_B._conradae         .......... ........-. .......... .......... .......... T-........ ....-..... ..--.....- -------A.- ----------
AY998763_B.duncani_W2        .......... ........-. .C........ .......... ....GC..T. .-........ G...--AC.. ......TT-- ---------- ----------
AY998762_B._duncani_W1       .......... ........-. .C........ .......... ....GC..T. .-........ G...--AC.. ......TT-- ---------- ----------
AY998769_Babesia_sp._RD1     .......... A.......-. .......... .......... .....CCC.. .-........ G...-AAC.A ....CCAT-- ---------- ------GGCA
AY998765_Babesia_sp._CA1     .......... A.......-. .......... .......... .....CCC.. .-........ G...-AAC.A ....CCAC-- ---------- ------GGCA
AY965740_B._duncani_CA6      .......... ........-. .C........ .......... ....GC..T. .-........ G...--AC.. ......TT-- ---------- ----------
AY998761_B.duncani_CA5       .......... ........-. .C........ .......... ....GC..T. .-........ G...--AC.. ......TT-- ---------- ----------
AB112337_B._microti_GI       ......T... ....T.C.-. .A..A..G.A .......... C.CCTC.... .A.......G -----TATAC C..TT.CCAA GTTATGC.T. GTGAGTGGTG
AF510203_B._microti          ......T... ....T..TT. ....A..G.A .......... C.CCT..... .A........ -----T-TA. A..TT.ATAT AAT----.T. GTGAGTGATA

                                    210        220        230        240        250        260        270        280        290        300
                             ....|....| ....|....| ....|....| ....|....| ....|....| ....|....| ....|....| ....|....| ....|....| ....|....|
GQ411430_B._lengau           ---------- -GGCTTTTGT GATTGGGTAT CTCATG--TT GAGTACCTGA ATTTCCA-AA GCGAGATTCT AGTCGTGGTG TGCGACGGAG GTTTCCATTT
our_sequences                ---------- -......... .......... ...C.TGTGC ........T. .G...A.TC. A......GT- ---TCC...A C.T...A.C. ...CA.C---
AY965739_B._conradae         ---------- -..T.....A ..G...A.G. A..T.---.. ...C.T..C. ......GC.. ......G..- GCCT.....T ..GCGG.A.. T..GGTC.C.
AY998763_B.duncani_W2        ---------- T....C.... ........G. ...T.---.. ....G...T. ......GTTT ......G.T- ----.CC.AT G...G..... .C..TGC---
AY998762_B._duncani_W1       ---------- T....C.... ........G. ...T.---.. ....G...T. ......GTTT ......G.T- ----.CC.AT G...G..... .C..TGC---
AY998769_Babesia_sp._RD1     CTTGGTTCCG -........C .......CGG ...T.---G. ...CG...C. ...C..GC.. ......GGA- ----.CCC.A G..TG..... .G..TTCGG-
AY998765_Babesia_sp._CA1     CTTGGTTCCG -........C .......CGG ...T.---G. ...CG...C. ...C..GC.. ......GGA- ----.CCC.A G..TG..... .G..TTCGG-
AY965740_B._duncani_CA6      ---------- T....C.... ........G. ...T.---.. ....G...T. ......GTTT ......G.T- ----.CC.AT G...G..... .C..TGC---
AY998761_B.duncani_CA5       ---------- T....C.... ........G. ...T.---.. ....G...T. ......GTTT ......G.T- ----.CC.AT G...G..... .C..TGC---
AB112337_B._microti_GI       CGCAACAG-G TTTTC.G... .TG------- -CAT.TAAG. TG..GTGACT GACG.GTTT. T.AC.CAGAA G..TC----- ---------- ---.TTGAAA
AF510203_B._microti          TACAA----A TTTTA.G.A. AT.------- -.A.GT--G. T....A..TT GAGCTG.ATC T.AGATAAAA ...TC----- ---------- ---.TTGAAA

                                    310        320        330        340        350        360        370        380        390        400
                             ....|....| ....|....| ....|....| ....|....| ....|....| ....|....| ....|....| ....|....| ....|....| ....|....|
GQ411430_B._lengau           GGG-----TA ACCTTGATCT CGGAGCTTCT TGTTGTTTAA TTATAAACTT GATGCTTCCT T--GTAGGTC CT-TAACCTG TGTAT----- --ACAGGTTT
our_sequences                ---------- -GTGA..... ...G..ACT. ..A.A.A--- ---.TT.T.. A.....C.T. G----..A.T TGGC.TA..A .T.T.TTTTT GA...TTA.C
AY965739_B._conradae         A..GACTT.. CTT.C..... .......... ......A..- .AT.CT.... .......... .--....... .CA.CTT..A ..C..TAGTA TAGT..A.C.
AY998763_B.duncani_W2        ---------- --........ ...G..C... .......--G ....-G.... .......... .--....... .G-G.CTGGA AACGGTCTAG CGCA.CCGCG
AY998762_B._duncani_W1       ---------- --........ ...G..C... .......--G ....-G.... .......... .--....... .G-G.CTGGA AACGGTCTAG CGCA.CCGCG
AY998769_Babesia_sp._RD1     ------A--- C......... .......... ......AG.. A..C-T...C ..ATGC.T.C .TGAAG.TC. .G-GCT.TGC C.AGCGCGTC CG.GGTTCCA
AY998765_Babesia_sp._CA1     ------A--- C......... .......... ......AG.. A..C-T...C ..ATGC.T.C .TGAAG.TC. .G-GCT.TGC C.AGCGCGTC CG.GGTTCC.
AY965740_B._duncani_CA6      ---------- --........ ...G..C... .......--G ....-G.... .......... .--......M .G-G.CTGGA AACGGTCTAG CGCA.CCGCG
AY998761_B.duncani_CA5       ---------- --........ ...G..C... .......--G ....-G.... .......... .--....... .G-G.CTGGA AACGGTCTAG CGCA.CCGCG
AB112337_B._microti_GI       CTTGGA---C TTG..TG.G. G.T..A.AG. .TAGT.AGCC GCGC.GCG.A ...A------ -------.AA A.ACGT---- -----TGTTG ATGTT--...
AF510203_B._microti          CTTGGA---C TTG..T..T. G-T..A-ATC .CAAC.C.T. ...C------ ---------- ---------- ---C.T---- -----TATTG G------...

                                    410        420        430        440        450        460        470        480        490        500
                             ....|....| ....|....| ....|....| ....|....| ....|....| ....|....| ....|....| ....|....| ....|....| ....|....|
GQ411430_B._lengau           -TGCGTCGGC ATT----ACC TAGCAG---- ---------- ---------- ---------- ---------- ---------- ---------- ----------
our_sequences                TA.TT.TT.T .A.----GTT .CCTTAATAT TGACTTGCGT ---------- ---------- ---------- ---------- ---------- ----------
AY965739_B._conradae         G....C.AC. ..A----.T. GTT.GAGCTT CAAATTTAGG TT-------- ---------- ---------- ---------- ---------- ----------
AY998763_B.duncani_W2        GATATCTT.A GCA----G.T A.ATGTGCTT TATTTCTCTT CCTGAAATTG GGTGAGGCTA TCCGCTGAAT TTAAGC---- ---------- ----------
AY998762_B._duncani_W1       G.TA.CTT.A GCA----G.T A.-TGTGCTT TGTTTCTCTT CCTGAAATTG GGTGAGACTA TCCGCTGAAT TTAAGC---- ---------- ----------
```

```
AY998769_Babesia_sp._RD1  GGAGC.TTTA GGC----GT. AGC...GCT- ---------- ---------- ---------- ---------- ---------- ---------- ----------
AY998765_Babesia_sp._CA1  GGAGC.TTTA GGC----GT. AGC...GCTT TTGTTCCTGA AATTGGGTGA GGCTATCCGC TGAATTTAAG C--------- ---------- ----------
AY965740_B._duncani_CA6   G.TA.CTT.A GCA----G-- ---------- ---------- ---------- ---------- ---------- ---------- ----------
AY998761_B.duncani_CA5    G.TA.CTT.A GCA----G.T A.-TGTG--- ---------- ---------- ---------- ---------- ---------- ---------- ----------
AB112337_B._microti_GI    T.AT.G.--- ---------- ---.T.AAAT TGGATGTGAT GATCCGCTGA ATTTAAGCAT ATAACTAAGC GGAAGAAAAG AAAATAACAA TGATTCCCTT
AF510203_B._microti       T.TT.G.--- ---------- ---.T.AA-T TGGATGTGAC GATCCGCTGA ATTTAAGCAT ATAACTAAGC GGAAGAAAAG AAAATAACTA TGATTCCCTC


                          .
GQ411430_B._lengau
our_sequences             -
AY965739_B._conradae      -
AY998763_B.duncani_W2     -
AY998762_B._duncani_W1    -
AY998769_Babesia_sp._RD1  -
AY998765_Babesia_sp._CA1  -
AY965740_B._duncani_CA6   -
AY998761_B.duncani_CA5    -
AB112337_B._microti_GI
AF510203_B._microti       -
```
