# Supplementary material for: Babesia behnkei sp. nov., a novel Babesia species infecting isolated populations of Wagner’s gerbil, Dipodillus dasyurus, from the Sinai Mountains, Egypt
Source: Parasit Vectors. 2014 Dec 9;7:572. doi: 10.1186/s13071-014-0572-9 (PMC4271447; doi:10.1186/s13071-014-0572-9)
Supplement: Additional file 3: — Alignment of the ITS1 region. [file 13071_2014_572_MOESM3_ESM.pdf]

```
                                       10         20         30
                             ....|....| ....|....| ....|....| ....|.
KM067276_Babesia_behnkei     TGTGTGAGCC AAGACATCCA TCGCT-GAAA GTTTAA
AF158702_Babesia_conradae    CA..GA.TAA T.A.GTAGG. CTTTGGTTCT A...TG
AY027815_Babesia_sp._WA1     CA..GA.TAA T.A.GTAGG. CTTTGGTTCT A...TG
HQ264129_Babesia_bovis       .AG....A.. TGCGG.AGG. ..AT--TC.C A.----
DQ200887_Babesia_poelea      CA..GA.TAA T.A.GTAGG. CTTTGGTTCT A...TG
DQ287951_Babesia_equi        CA..GA.TAA TG..GTAGG. CTTTGGTTCT A...TG
HM113372_Babesia_sp._EU1     CA..GA.TAA T...GTAGG. CTTTGGTTCT A...TG
AB112337_Babesia_microti     .AG....A.. TGCGG.AGG. ..AT--TCTT A.CAG.
AB190435_Babesia_microti     CA..GA.TAA TGA.GTAGG. CTTTGGTTCT A...TG
AF510200_Babesia_microti     .AG....A.. TGCGG.AGG. ..AT--TCTT A..AG.

                                       90        100        110
                             ....|....| ....|....| ....|....| ....|.
KM067276_Babesia_behnkei     GTTGACAAAT AATATCCACA AAAGATATA- ---TAA
AF158702_Babesia_conradae    .CATT.GT.. TTA.CTGT.. G.G.TG.A.T TCT..G
AY027815_Babesia_sp._WA1     .CATT.GT.. TTA.CTGT.. G.G.TG.A.T TCT..G
HQ264129_Babesia_bovis       AAGCGA.GC. TCC.C.---- ---AGAGC.C TC----
DQ200887_Babesia_poelea      .CATT.GT.. TTA.CTGT.. G.G.TG.A.T TCT..G
DQ287951_Babesia_equi        .CATT.GT.. TTG.CTGT.. G.G.TG.A.T TCT..G
HM113372_Babesia_sp._EU1     .CATT.GT.. TTA.CTGT.. G.G.TG.A.T TCT..G
AB112337_Babesia_microti     .G.CG..CC. TC..C.TT.T .G.AGAG.GG CCT.GG
AB190435_Babesia_microti     .CATT.GT.. TTA.CTGT.. G.G.TG.A.T TCT..G
AF510200_Babesia_microti     .G.CG..CC. TT...----- --..TAG.GG CCT.GG

                                      170        180        190
                             ....|....| ....|....| ....|....| ....|.
KM067276_Babesia_behnkei     CTGG---TGG TCAGTACACA ACTTGAAAC- ---CAG
AF158702_Babesia_conradae    T.TCATTAAT CA..A..GA. .G..AGGGGC T--.GA
AY027815_Babesia_sp._WA1     T.TCATTAAT CA..A..GA. .G..AGGGGC T--.GA
HQ264129_Babesia_bovis       .A-----AC. C.G.CTAC.C TAG.AGCCGG T--TG.
DQ200887_Babesia_poelea      T.TCATTAAT CA..A..GA. .G..AGGGGA T--.GA
DQ287951_Babesia_equi        T.CCATTAAT CA..A..GA. .G..AGGGGA T--.GA
HM113372_Babesia_sp._EU1     T.CCATTAAT CA..A..GA. .G..AGGGGA T--.GA
AB112337_Babesia_microti     .GCAAGCGC. ..G.A.GG.G GG..TGTCTG C-A.G.
AB190435_Babesia_microti     T.TCATTAAT CA..A..GA. .G..AGGGGA T--.GA
AF510200_Babesia_microti     .ACAA-CGC. ..G.A.GG.G GG..TGTC.G CTATG.

                                      250        260        270
                             ....|....| ....|....| ....|....| ....|.
KM067276_Babesia_behnkei     GCATA-ACCC GAATGTTAAT TATAGAATAA CATCGG
AF158702_Babesia_conradae    CT.G.G.TTG ..GGTCGTCA .T.TA..CG. .TC.TT
AY027815_Babesia_sp._WA1     CT.G.G.TTG ..GGTCGTCA .T.TA..CG. .TC.TT
HQ264129_Babesia_bovis       ------..TG CC.C.ACCC- ----.GG.T. AGCTC.
DQ200887_Babesia_poelea      CT.G.G.TTG ..GGTCGTCA .TGTA..CG. .TC.TT
DQ287951_Babesia_equi        CT.G.G.TTG ..GGTCGTCA GT.T...CG. .TC.TT
```

```
HM113372_Babesia_sp._EU1 CT.GGG.TTG ..GGTCGTCA .T.TTC-CG. .TC.TT
AB112337_Babesia_microti ATC---.TT. T..C.GGC.. .GGG.G..T. T.CT.T
AB190435_Babesia_microti CT.G.G.TTG ..GGTCGTCA GT.TA..CG. .TC.TT
AF510200_Babesia_microti ATC---.TT. T..C.GGCT. --GG.GGCT. T.CT.T

                                  330        340        350
                         ....|....| ....|....| ....|....| ....|.
KM067276_Babesia_behnkei CAGAGATTTG TTTGGCCTTG AACGTATCCA AGTGCC
AF158702_Babesia_conradae GG.GAG.A.. G.C.CAAGGC TGAAAC.TA. ..GAAT
AY027815_Babesia_sp._WA1 GG.GAG.A.. G.C.CAAGGC TGAAAC.TA. ..GAAT
HQ264129_Babesia_bovis   GGTGCC...C A..CC----- ---AGCC.TT TAG.G.
DQ200887_Babesia_poelea  GG.GAG.A.. G.C.CAAGGC TGAAAC.TA. ..GAAT
DQ287951_Babesia_equi    GG.GAG.A.. G.C.CAAGGC TGAAAC.TA. ..GAAT
HM113372_Babesia_sp._EU1 GG.GAG.A.. G.C.CAAGGC TGAAAC.TA. ..GAAT
AB112337_Babesia_microti .GTGAC...T G.CTC.GGAT ..G.GTC.AT GCGAG.
AB190435_Babesia_microti GG.GAG.A.. G.C.CAAG.C TGAAAC.TA. ..GAAT
AF510200_Babesia_microti .GTG.C...T C.C.------ G.A.GTCATG ..GAG.

                                  410        420        430
                         ....|....| ....|....| ....|....| ....|.
KM067276_Babesia_behnkei TCTTGCAACG ACAAAAAGCT CTGT------ ---CGC
AF158702_Babesia_conradae .GACT....A CGGGG..C.. .ACCAGGTCC AGA.AT
AY027815_Babesia_sp._WA1 .GACT....A CGGGG..C.. .ACCAGGTCC AGA.AT
HQ264129_Babesia_bovis   .--CC..G.. .TGG.TGC.. .G.CTCGCGC C-T..A
DQ200887_Babesia_poelea  .GACT....A CGGGG..A.. .ACCAGGTCC AGA.AG
DQ287951_Babesia_equi    .GACT....A CGGGG..A.. .ACCAGGTCC AGA.AG
HM113372_Babesia_sp._EU1 .GACT....A CGGGG..A.. .ACCAGGTCC AGA.AA
AB112337_Babesia_microti .--.T..G.. GTGG.TGT.. .G.CTCACAC A-A..A
AB190435_Babesia_microti .GACT....A CGGG...C.. .ACCAGGTCC AGA.AT
AF510200_Babesia_microti .--.T..G.. GTGG.TGT.. .G.CTCACAC A-A..A

                                  490        500        510
                         ....|....| ....|....| ....|....| ....|.
KM067276_Babesia_behnkei -CCAATAAAC CGGTACCGCC TTTTTTAAAC CATAAA
AF158702_Babesia_conradae AGTGG.GC.T G.CCG--TT. ..AG..GGTG G.GTG.
AY027815_Babesia_sp._WA1 AGTGG.GC.T G.CCG--TT. ..AG..GGTG G.GTG.
HQ264129_Babesia_bovis   T.TTGCG.-T T.C..--.A. C.-C.G..CG T.ACC.
DQ200887_Babesia_poelea  GGTGG.GC.T G.CCG--TT. ..AG..GGTG G.GTG.
DQ287951_Babesia_equi    GGTGG.GC.T G.CCG--TT. ..A...GGTG G.GTG.
HM113372_Babesia_sp._EU1 GGTGG.GC.T G.CCG--TT. ..AG..GGTG G.GTG.
AB112337_Babesia_microti TTT.GC...T .AAC.--.GT ..-C.G..TG T..TGT
AB190435_Babesia_microti GGTGG.GC.T G.CCG--TT. ..AG..GGTG G.GTG.
AF510200_Babesia_microti TTT.GC...T .AAC.--.GT ..-C.G..TG T..TGT

                                  570        580        590
                         ....|....| ....|....| ....|....| ....|.
KM067276_Babesia_behnkei GTTACGA--- ----CTTCTC CTTCCTTTAA GTGATA
```

```
AF158702_Babesia_conradae  .C..AAT-AG CAGCTGAGAA TAAA.-..TG T.--GT
AY027815_Babesia_sp._WA1   .C..AAT-AG CAGCTGAGAA TAA---.CTC T.--GT
HQ264129_Babesia_bovis     T.CCA.---- TATGGCA.AA TGC.GCCATG .CAGGG
DQ200887_Babesia_poelea    .C..AAT-AG CGGC.GAGAA TAAA....TG T.--.G
DQ287951_Babesia_equi      .C..AAT-AG GATG.GAGA- ------..TG -----G
HM113372_Babesia_sp._EU1   .C..ACT-AG TACC.G.AAA AAGG--..CG TC--CG
AB112337_Babesia_microti   C...AA.TCC TAAAG.ATA. ...TT.CC.. ..T..G
AB190435_Babesia_microti   .C..AATTAG GATCTGGGA. AAG.--..TG C.---G
AF510200_Babesia_microti   C...AA.TCC TAAAG.ATA. ...TT.CC.. ..-..G

                                    650        660        670
                           ....|....| ....|....| ....|....| ..
KM067276_Babesia_behnkei   CACGGTCCGA ATAATTCACC GGATCACTCG A-
AF158702_Babesia_conradae  ---------- ---------- ---------- --
AY027815_Babesia_sp._WA1   ---------- ---------- ---------- --
HQ264129_Babesia_bovis     ATA.A----- ---------- ---------- --
DQ200887_Babesia_poelea    ---------- ---------- ---------- --
DQ287951_Babesia_equi      ---------- ---------- ---------- --
HM113372_Babesia_sp._EU1   ---------- ---------- ---------- --
AB112337_Babesia_microti   GTT...GT.. C.-GACGCGT TT.....G.A G-
AB190435_Babesia_microti   ..A..AAGTG TA.GGCA.TA AC.GGT..GT GT
AF510200_Babesia_microti   GTT...GT.. C..GACGCGT TT.....G.A G-
```
